# Relationships among cognition, emotion, and motivation: implications for intervention and neuroplasticity in psychopathology

**DOI:** 10.3389/fnhum.2013.00261

**Published:** 2013-06-11

**Authors:** Laura D. Crocker, Wendy Heller, Stacie L. Warren, Aminda J. O'Hare, Zachary P. Infantolino, Gregory A. Miller

**Affiliations:** ^1^Department of Psychology, University of Illinois at Urbana-ChampaignChampaign, IL, USA; ^2^Department of Mental Health, St. Louis VA Medical CenterSt. Louis, MO, USA; ^3^Department of Psychology, University of Massachusetts DartmouthNorth Dartmouth, MA, USA; ^4^Department of Psychology, University of DelawareNewark, DE, USA; ^5^Department of Psychology, University of KonstanzKonstanz, Germany

**Keywords:** emotion, cognition, motivation, anxiety, depression, neuroplasticity, intervention

## Abstract

Emotion-cognition and motivation-cognition relationships and related brain mechanisms are receiving increasing attention in the clinical research literature as a means of understanding diverse types of psychopathology and improving biological and psychological treatments. This paper reviews and integrates some of the growing evidence for cognitive biases and deficits in depression and anxiety, how these disruptions interact with emotional and motivational processes, and what brain mechanisms appear to be involved. This integration sets the stage for understanding the role of neuroplasticity in implementing change in cognitive, emotional, and motivational processes in psychopathology as a function of intervention.

## Introduction

Research on emotion and its relationship with cognition has garnered much attention in recent years (e.g., Phelps, 2006; Levin et al., 2007; Pessoa, 2008; Miller, 2010; Dolcos et al., 2011), evident in the increasing popularity of the term “emotion-cognition interactions” in the literature. This body of literature has come to appreciate the intimate and closely interacting nature of these processes and is expanding to understand the relationships between motivational and cognitive processes (Spielberg et al., 2008, 2011b; Pessoa, 2009; Pessoa and Engelmann, 2010; Chiew and Braver, 2011). We define emotion as a system of multiple related processes (including relevant thoughts, experiences, and preparations for action, manifesting in physiology, overt behavior, and language; Lang, 1968; Kozak and Miller, 1982; Roseman, 2008) that “attempt to promote adaptation by responding to the pursuit and attainment (or lack of attainment) of individuals' needs, goals, and concerns” (Berenbaum et al., 2003, pg. 208). Similar to cognition and emotion, emotion and motivation are related constructs but are not identical (for further discussion, see Chiew and Braver, 2011). Although emotions and motivations both have a hedonic component, motivations are typically conceptualized as processes that drive goal-directed behaviors aimed at achieving desired outcomes and avoiding undesired ones (Carver, 2006; Roseman, 2008). Pessoa (2009) described motivation as “what makes one work to obtain a reward or to avoid punishment.”

Although some have argued that emotions and motivations cannot be separated (Buck, 2000; Laming, 2000), many argue that these constructs are related yet distinguishable, and differ in their effects on cognition and behavior (for a review, see Chiew and Braver, 2011). These psychological processes are implemented via both shared and distinct brain regions. Carver (2006) proposed that emotion is the affect that emerges from comparing the actual versus expected progress toward a goal, whereas motivation is what drives progress toward that goal. When there is a mismatch between actual and expected progress, changes in emotional states occur and alter subsequent motivations, impeding or promoting goal attainment. Further, changes in motivation may modify expectations about future events, which can then result in changes in emotions.

Accumulating evidence demonstrates that performance on tasks commonly considered nonemotional can be influenced by emotional and motivational states, more enduring emotion- and motivation-related traits, and the emotional qualities of situations. Cognitive processing is also an integral part of emotion and motivation and affects the degree to which they influence ongoing activities and behaviors. It has become increasingly clear that cognition, emotion, and motivation are intricately intertwined, and it is difficult to determine where to draw the line between them (Pessoa, 2008, 2009; Miller, 2010). Complex relationships among these psychological processes appear to play an important role in the development and maintenance of psychopathology and in treatment effectiveness. As demonstrated below, a review of the cognitive difficulties experienced by individuals with anxiety and depression makes clear that it is virtually impossible to separate these difficulties from their emotional and motivational influences. Conversely, the emotional and motivational disruptions that are characteristic of anxiety and depression are embedded in abnormal cognitions, as has been well established for some time (e.g., Beck, 1976; Levin et al., 2007) and can be targeted effectively in treatment paradigms.

Recent years have also seen advances in elucidating the functional and structural brain mechanisms that support the effects of emotion and motivation on cognition and vice versa (for reviews, see Gray, 2004; Phelps, 2006; Pessoa, 2008, 2009; Chiew and Braver, 2011; Dolcos et al., 2011; Ochsner et al., 2012). Researchers have often used neuroimaging data to support the notion of functional specialization in the brain, carving it into distinct “cognitive,” “affective,” and “motivational” regions. Growing sophistication in theory and methodological approaches has led to empirical evidence suggesting that these processes are not only interdependent but effectively integrated in at least some areas of the brain (e.g., Gray, 2004; Pessoa, 2008, 2009; Miller, 2010). Cognitive, emotional, and motivational processes are implemented by overlapping networks of regions that play various roles depending on the task/context. These networks include prefrontal cortex (PFC), cingulate, amygdala, striatum, hypothalamus, hippocampus, insula, and parietal regions. Despite a growing body of research on this topic, much work remains to be done, especially to advance concepts and theories to guide the work (Miller, 1996, 2010). There continues to be enormous but unrealized potential to apply these findings to psychopathology and treatment (Miller et al., 2007; Carrig et al., 2009; Fu et al., 2012). A better understanding of the psychological and neural mechanisms involved in the complex relationships between cognition, emotion, and motivation can aid in advancing the development of such new applications.

The goals of this paper are (1) to integrate findings of studies exploring relationships between cognitive, emotional, and motivational processes, and their associated neural mechanisms in anxiety and depression and (2) to highlight psychological and biological processes implicated in emotion-cognition and motivation-cognition interactions that are amenable to ongoing modification and can be targeted with interventions. Thus, this review will convey the current state of the field and highlight the potential synergy between basic and treatment-related research that can move the field forward.

In the present review, neuroplasticity refers to functional and structural flexibility of brain systems, regions, and structures over time, such that a given system is able to change in response to input (which may include experience or other interventions) and does not harden into rigidity with maturation. In some cases a functional change might reflect alterations in dynamic neural processes as inferred by modifications in activity and metabolism or other aspects of physiology. In such cases there is no presumption that the altered physiology directly influences or reflects change in the structure of the neural tissue. In other cases, the neural tissue itself may be the substrate of neuroplasticity inasmuch as there is alteration in cellular and/or regional structure. The present review will focus on anxiety and depression, but manifestations of other types of psychopathology are also highly dependent on emotion-cognition and motivation-cognition interactions. For example, the clinical picture of schizophrenia is influenced significantly by emotional adjustment, motivational dynamics (e.g., reward responsivity, approach/avoidance coping style), and cognitive biases, each of which interacts with the other and the clinical course of the disorder (e.g., Rector and Beck, 2002). Explication of the dynamics of emotion-cognition and motivation-cognition processes in anxiety and depression may contribute to understanding similar dynamics in other disorders.

## Emotion-cognition interactions in anxiety and depression

Emotion-cognition interactions gone awry can lead to clinically significant levels of anxiety and depression. For example, anxiety and depression are characterized by information-processing biases and cognitive dysfunction, which appear to contribute to the onset and/or maintenance of symptoms, including persistent negative affect and poor emotion regulation. A pervasive finding in the anxiety literature is that anxious individuals exhibit an attentional bias, such that they preferentially process threat-related information (for reviews, see McNally, 1998; Bar-Haim et al., 2007). Anxious individuals display facilitated orientation toward threatening stimuli and have difficulty disengaging from it once their attention is captured (for reviews, see Cisler et al., 2009; Sass et al., 2010). This attentional bias appears to play a key role in the etiology and maintenance of anxiety disorders (MacLeod et al., 2002; Amir et al., 2009; Koster et al., 2009).

There is also some evidence that depressed individuals exhibit an attentional bias to negative material, though this literature is mixed (for reviews, see Levin et al., 2007; Gotlib and Joormann, 2010). When biased attention has been found in depression, it has often been the case that stimuli were presented for relatively longer durations (e.g., >500 ms, see Bradley et al., 1997; Gotlib et al., 2004) than is typical in the anxiety literature, in which stimuli are presented very briefly (e.g., <100 ms). Williams and colleagues (1997) proposed that the attentional biases for threat observed in studies of anxiety reflect earlier stages of processing (e.g., orienting), whereas biases in depression reflect later stages of processing (e.g., elaborative processing). However, some scalp event-related brain potential (ERP) findings have indicated a bias to attend to negative words as early as 200 ms post stimulus onset, as well as later enhanced processing in depression with comorbid anxiety (Sass et al., under review). Thus, evidence suggests that impairments in control of attention, particularly in the face of distracting emotional information, characterize both depression and anxiety, although potentially in different ways or on different time scales.

Hemodynamic neuroimaging work examining the successful implementation of control of attention in the context of emotional distractors has implicated several key areas, including dorsolateral prefrontal cortex (DLPFC) and anterior cingulate cortex (ACC; Whalen et al., 1998; Compton et al., 2003; Mohanty et al., 2007; Banich et al., 2009; Herrington et al., 2010). Not surprisingly, these areas appear to function abnormally in anxiety and depression, such that dysfunction in DLPFC, as well as in dorsal ACC (dACC) and rostral ACC (rACC), has been associated with difficulty ignoring distracting emotional information (e.g., Engels et al., 2007, 2010; Bishop, 2008; Herrington et al., 2010). Additionally, various parts of the parietal cortex play a role in control of attention in both emotional and nonemotional contexts (Banich et al., 2000; Compton et al., 2003; Corbetta et al., 2008) and are disrupted in anxiety and depression (Bruder et al., 1997; Engels et al., 2007; for reviews, see Heller, 1993; Heller et al., 2003). Together, these findings suggest that anxiety and depression are associated with abnormal cognition in the presence of emotional distractors, from earlier selective attention to later inhibition and response selection.

There is ample evidence that anxious individuals also exhibit an interpretation bias, in which ambiguous information and situations are interpreted negatively (Mathews and MacLeod, 2005; Zinbarg and Yoon, 2008). This bias is supported by two fMRI findings (for a review, see Bishop, 2007). First, responsivity of the amygdala to neutral stimuli increases as a function of anxiety, suggesting that anxious individuals overinterpret such stimuli as threatening (Somerville et al., 2004). Second, PFC is engaged when healthy individuals attempt to decrease the impact of negative information via emotion-regulation strategies, including generating new interpretations of situations. Individuals with anxiety exhibit decreased PFC recruitment during such tasks, suggesting that they have difficulty generating alternative meanings of such stimuli in order to alter their initial and ongoing emotional response (Goldin et al., 2009). This interpretation bias appears to play a causal role in anxiety and can lead to distortions in memory (Wilson et al., 2006; Hirsch et al., 2009; Hertel and Brozovich, 2010).

It is unclear whether depression is also associated with an interpretation bias, given mixed results in the literature (for discussion, see Gotlib and Joormann, 2010). However, there is consistent evidence that depression is characterized by a memory bias, such that depressed individuals preferentially recall negative over positive information (for review, see Mathews and MacLeod, 2005; Gotlib and Joormann, 2010). Depressed individuals also tend to retrieve overgeneral autobiographical memories that lack details, even when they are instructed to recall specific events (Williams et al., 2007). Consistent with these findings, hypoactivation of the hippocampus and parahippocampal gyrus has been observed in individuals diagnosed with major depressive disorder (MDD) during an autobiographical memory task (Young et al., 2012). Given deficits in DLPFC activation in depressed individuals, difficulty implementing strategies to recall detailed memories may be related to impaired connectivity between PFC and hippocampal regions. Overgeneral memory recall has been associated with longer depressive episodes (Raes et al., 2005), delayed recovery from affective disorders (Dalgleish et al., 2001), and less complete recovery from major depression (Brittlebank et al., 1993).

## Executive function deficits in anxiety and depression

Anxiety and depression have been associated with deficits in executive function (EF; Levin et al., 2007; Snyder, 2013; Snyder et al., under review), which may contribute to the observed emotion-cognition problems reviewed above. EF can be defined as the “set of abilities required to effortfully guide behavior toward a goal, especially in nonroutine situations” (Banich, 2009, p. 89). Examples of EFs include planning and organizing, sequencing steps to accomplish a task, inhibiting prepotent responses, updating and manipulating information in working memory, shifting between strategies or tasks, and flexibly adjusting behavior to environmental demands. A pervasive view in the literature is that the EF deficits that characterize anxiety and depression are due to the symptoms of psychopathology (e.g., Williams et al., 2000; Eysenck et al., 2007) and hence resolve when symptoms remit. However, others have asserted that these deficits are not simply the result of current symptoms, and several studies have demonstrated that individuals in remission from depression still exhibit various EF deficits (Beats et al., 1996; Paradiso et al., 1997; Austin et al., 2001). Given that executive dysfunction persists even when symptoms improve, it is plausible that these EF deficits contribute to initial onset or relapse, rather than merely resulting from disorder.

There is evidence that anxiety is associated with deficits in shifting between mental sets (Airaksinen et al., 2005; Johnson, 2009), although others have failed to replicate this finding (Castaneda et al., 2010). In addition, anxiety has been linked to working memory problems (MacLeod and Donnelan, 1993; Derakshan and Eysenck, 1998; Eysenck et al., 2005), particularly under stressful conditions (Eysenck et al., 2007). An influential proposal, the attentional control theory, considers anxiety in relation to three EF components—inhibition, shifting, and updating of working memory—based on a model proposed by Miyake and colleagues (2000). This theory proposes that anxiety is characterized by an EF deficit in control of attention due to worry impairing the central executive of the working memory system (Eysenck et al., 2007). This impairment is accompanied by deficits in inhibition and shifting functions, as well as an imbalance in two attention systems. Specifically, anxiety decreases the influence of a goal-directed, top-down attention system and increases the influence of a stimulus-driven, bottom-up attention system. Little work has been conducted thus far investigating key aspects of this theory, but some support of its assertions is starting to accrue (for reviews, see Derakshan and Eysenck, 2009; Eysenck and Derakshan, 2011; Snyder et al., under review).

Using the three-component EF model developed by Miyake and colleagues (2000), Warren et al. (under review) found that the specificity of anxiety-related EF impairments depended on differentiating dimensions of anxiety, specifically anxious apprehension or worry from anxious arousal or sympathetic hyperarousal. Whereas anxious apprehension was associated with shifting impairments only, anxious arousal was associated with broad impairments in EF (shifting, updating, and inhibition), especially updating and inhibition. These findings are generally consistent with Eysenck et al.'s (2007) prediction that anxiety impairs shifting and inhibition, although they extend the attentional control theory to suggest that distinct dimensions of anxiety are associated with specific patterns of executive dysfunction (Warren et al., under review). Future work should examine these dimensions of psychopathology in relation to Miyake and Friedman's (2012) updated EF model in which the inhibition-specific component is subsumed by a common EF factor. This factor is what is common across all 3 EFs (inhibition, shifting, and updating) and may reflect the ability to “actively maintain task goals and goal-related information” (Miyake and Friedman, 2012, p.11).

Deficits in inhibition appear to be associated with the difficulties that depressed individuals have disengaging from mood-congruent negative information, which leads to further elaboration of the negative information and contributes to the attentional bias described above (for a review, see Gotlib and Joormann, 2010). Some evidence suggests that this effect is valence-specific, such that depressed individuals demonstrate inhibition deficits selectively for negative information (e.g., Goeleven et al., 2006). In addition, depressed individuals have difficulty intentionally ignoring distracting information, whether it is emotional or nonemotional in nature (Gotlib and Joormann, 2010; Snyder, 2013). Depression therefore appears to be associated with an increased vulnerability to distracting information, but once attention has been captured, difficulties in disengaging are specific to information with negative valence.

Depression-related difficulty disengaging from information also appears to be related to deficits in other cognitive control mechanisms, specifically updating and removing previous task-relevant information, both emotional and nonemotional in nature, from working memory and flexibly switching attention to the task at hand (Joormann and Gotlib, 2008; Banich et al., 2009; Joormann, 2010; Warren et al., under review). These deficits likely also contribute to prolonged processing of negative aspects of stimuli, which in turn hinders emotion regulation processes and leads to the sustained negative affect and rumination observed during depressive episodes (Joormann, 2010). Further, depression has been associated with a variety of other EF deficits, including impairments in verbal fluency, verbal and visuospatial working memory, and planning (for reviews, see Yee, 1995; Levin et al., 2007; Snyder, 2013).

Studies of healthy individuals have consistently implicated several subregions of PFC across a variety of EFs. Specifically, DLPFC, ventrolateral PFC (VLPFC), and dACC are recruited during tasks involving inhibition, shifting, working memory, and planning (Wager and Smith, 2003; Wager et al., 2004; Collette et al., 2005, 2006; Nee et al., 2007). Depression and anxiety have both been associated with hypoactivation in these regions (Rogers et al., 2004; Levin et al., 2007; Fitzgerald et al., 2008; Bishop, 2009). Impaired recruitment of PFC regions appears to be associated with difficulty implementing various functions associated with EF tasks, including maintaining task goals and goal-related information. Further, activity in left DLPFC has been shown to depend on levels of both anxiety and depression. Specifically, comorbid anxious arousal and depression were associated with reduced left DLPFC activity during an EF task, but only when anxious apprehension was low (Engels et al., 2010). In addition, anxiety and depression are associated with altered activity in a DLPFC-dACC network, albeit in distinct ways (Silton et al., 2011).

## Motivation-cognition interactions in anxiety and depression

Numerous behavioral and psychophysiological studies have provided evidence that depression is associated with motivation-related deficits. These are reflected in decreased responsivity to positive or rewarding stimuli and reduced approach-related behaviors (for reviews, see Fernandes and Miller, 1995; Pizzagalli et al., 2011). Relative to healthy controls, individuals with MDD exhibit blunted responsiveness to pleasant films and scenes (Berenbaum and Oltmanns, 1992; Sloan et al., 1997), to cues signaling the potential for reward (Pizzagalli et al., 2009a), and to receipt of actual rewards (Henriques and Davidson, 2000; Pizzagalli et al., 2009a). Depressed individuals also fail to demonstrate the bias toward attending and responding to positive and rewarding stimuli that nondepressed controls show (McCabe and Gotlib, 1995; Pizzagalli et al., 2009b).

Hemodynamic neuroimaging studies of reward tasks have demonstrated that depression is associated with decreased activation in key brain areas associated with the processing of reward-related information, specifically nucleus accumbens and caudate, as well as decreased activation in left PFC, an area that has been associated with approach-related motivation and the processing of positive stimuli (Davidson and Henriques, 2000; Herrington et al., 2005, 2010; Pizzagalli et al., 2009a; Wacker et al., 2009; Miller et al., 2013). Decreased activation in striatal areas has been found during both anticipatory and consummatory phases of reward processing (Pizzagalli et al., 2009a; Smoski et al., 2009). Other brain areas display abnormally increased activation in relation to reward processing in depression, including orbitofrontal cortex (OFC), implicated in the assessment of risk and reward, and dACC, implicated in predicting response value (Knutson et al., 2008; Smoski et al., 2009).

In addition to deficits in processing reward and decreased approach behavior, depression appears to be associated with increased avoidance behavior and an enhanced sensitivity to negative cues and punishment, consistent with a bias toward negative information as reviewed above (see also Pizzagalli et al., 2011). Furthermore, depressed individuals exhibit abnormal responses to errors and perceived failure and demonstrate problems adjusting their behavior appropriately after making mistakes and receiving negative feedback (Elliott et al., 1996, 1997; Heller and Nitschke, 1997; Murphy et al., 2003; Holmes and Pizzagalli, 2007, 2008). Studies examining brain activation in relation to the anticipation of and response to negative cues, feedback, and making errors have found hyperactivity in several areas associated with threat-related processing, including amygdala, ACC, and medial PFC (mPFC) along with hypoactivity in lateral PFC (Tucker et al., 2003; Holmes and Pizzagalli, 2008).

Although the literature on motivation and approach/avoidance behavior in psychopathology has typically focused on depression, there is evidence of abnormality in anxiety as well. Anxious individuals appear to be hypersensitive to negative or punishment-related stimuli, consistent with being prone to interpret information as threatening (for reviews, see Gray, 1975, 1982; Sass et al., 2010). Further, anxious individuals exhibit increased activation in threat-related brain regions when responding to negative stimuli, including PFC, dACC, amygdala, and parietal and temporal areas (Heller et al., 2003; Engels et al., 2007, 2010; Bishop, 2008; Olvet and Hajcak, 2008). Similar to depression, anxiety is associated with enhanced avoidance motivation (Spielberg et al., 2011a), such that anxious individuals habitually avoid potentially threatening situations (Barlow, 2002). The tendency for anxious individuals to engage in risk-avoidant behavior is due in part to exaggerated perceptions of the likelihood and cost of negative outcomes (Maner and Schmidt, 2006). Anxiety has been associated with increased activity in the insula while making risky decisions and learning to avoid monetary loss (Paulus et al., 2003; Samanez-Larkin et al., 2008; Damsa et al., 2009). The insula is a key brain area involved in both the experience and the anticipation of negative outcomes, as well as decision-making about risky behaviors (for a review, see Samanez-Larkin et al., 2008).

Furthermore, anxious individuals display hyper-reactivity to making errors, as evidenced by increased ACC activation and an enhancement in error-related negativity (ERN), an ERP component that indexes error processing (for a review, see Olvet and Hajcak, 2008). Anxiety also appears to be characterized by hypersensitivity to rewards, as it is associated with faster responses to potential rewards (Hardin et al., 2006) and increased activation in areas involved in reward processing (e.g., ventral striatum; Guyer et al., 2006, 2012; Bar-Haim et al., 2009). Thus, anxiety appears to be associated with exaggerated responses to both rewards and punishments, indicating enhanced sensitivity to incentives irrespective of valence.

It is likely that at least some of the observed motivation-related dysfunction associated with anxiety and depression is related to the EF deficits that also characterize these disorders. Adaptive motivational processing relies on intact EF, such that goals can be selected based on their predicted value, behaviors can be initiated to achieve these goals, and goal-directed action can be maintained across time, particularly in the face of distraction (Spielberg et al., 2012a,b). Many of the abnormal approach- and avoidance-related behaviors associated with anxiety and depression are likely due at least in part to dysfunction in specific EFs. For example, depressed individuals have difficulty sustaining reward responsiveness over time (Heller et al., 2009), which may be due to problems maintaining the contents of working memory, particularly when distractors are present (Yee and Miller, 1994). Heller and colleagues (2009) found that problems in reward responsiveness were linked to dysfunction in frontal and subcortical areas, which interact to implement goal-directed behavior.

Just as EFs appear to influence motivational processes, there is also evidence that motivation affects these cognitive processes in anxiety and depression. In healthy individuals, altering motivational processing via monetary incentives has been associated with enhancements of various EFs, including cognitive control, attention, set-shifting, and working memory (Pochon et al., 2002; Taylor et al., 2004; Engelmann and Pessoa, 2007; Engelmann et al., 2009; Jimura et al., 2010; Savine et al., 2010). In contrast, depressed adults and adolescents failed to adaptively adjust their performance during EF tasks in order to optimize their chances of winning money in rewarding and punishing contexts (Henriques and Davidson, 2000; Jazbec et al., 2005). Similarly, high trait-anxious individuals did not improve their performance during a demanding EF task when monetary incentives were offered, while low trait-anxious individuals demonstrated the expected enhanced performance in the reward condition (Eysenck, 1985). In a sample of anxious adolescents, incentive-related modulation of performance on a cognitive control task was significantly weaker than in healthy adolescents (Hardin et al., 2007).

The failure of motivational manipulations to appropriately modulate EFs in individuals with anxiety and depression is likely related to the observed dysfunction in brain networks associated with incentive processing and task-relevant cognitive processing. As reviewed above, anxiety and depression are associated with dysfunction in areas involved in processing both positive, rewarding stimuli and negative, punishing stimuli (e.g., putamen, caudate, and nucleus accumbens). Additionally, anxiety and depression have been associated with abnormal function in a network of brain regions involved in implementing EFs during various tasks, including DLPFC, dACC, rACC, and parietal cortex (Bruder et al., 1997; Heller et al., 2003; Engels et al., 2007, 2010; Bishop, 2008; Herrington et al., 2010). Furthermore, it is likely that networks involved in implementing motivation-related processes and EFs fail to interact appropriately in order to integrate various functions and successfully execute goal-driven behavior. Studies of healthy individuals have implicated several “hub” regions that link the two networks and integrate incentive-related processes with EFs: DLPFC, ACC, and posterior cingulate cortex (PCC; Pochon et al., 2002; Taylor et al., 2004; Locke and Braver, 2008; Pessoa, 2009; Jimura et al., 2010; Pessoa and Engelmann, 2010), all three of which have been associated with dysfunction in anxiety and depression (Bench et al., 1993; Mayberg, 1997; Mayberg et al., 1999; Engels et al., 2007, 2010; Bishop, 2008; Herrington et al., 2010).

## Relationships among EF, emotion, and motivation

Evidence reviewed above establishes many interactions among cognition, emotion, and motivation and clearly indicates that these interactions contribute to psychopathology. However, the mechanisms remain mostly speculative, and a question of interest concerns whether deficits in one domain predict or cause deficits in another, so as to affect the onset and/or maintenance of psychopathology. Although it is generally assumed that deficits in cognition and EF are caused by emotional and motivational disturbances, it has also been postulated that deficits in specific EFs (e.g., inhibition, shifting) are at least partly responsible for key cognitive, emotional, and motivational features of psychopathology, including cognitive biases, motivation-related dysfunction, and impaired emotion-regulation abilities (Levin et al., 2007; Gotlib and Joormann, 2010). For example, a bias to attend to negative information in anxious and depressed individuals may be driven in part by difficulties inhibiting distracting information or shifting attention to relevant aspects of a task. EFs may affect motivational processes, such that they alter ability to evaluate potentially pleasurable stimuli or activities or implement approach-related behaviors. EF deficits make it difficult to select goals based on their anticipated benefits and to implement strategies aimed at achieving these goals, particularly when distractions are present in the environment (Banich, 2009). EF deficits could also make it challenging for individuals to initiate and/or maintain emotion-regulation strategies aimed at promoting pleasant emotion or engaging adaptive coping behaviors that would buffer against the effects of stress (Monroe and Reid, 2009).

Some support for EF deficits contributing to emotion-related symptoms of psychopathology has been provided by recent research. Bredemeier and Berenbaum (in press) found that, when controlling for initial levels of worry, reduced working memory capacity predicted worry levels several weeks later. Similarly, research in our laboratory found that self-reported working memory difficulties predicted increases in symptoms of depression several months later, above and beyond the effects of initial depression (Letkiewicz et al., under review). Alexopoulos and colleagues (2000) found evidence that scores on measures of initiation and perseveration predicted early relapse, recurrence of depression, and the course of depressive symptoms post-remission. Interestingly, a treatment study of the response of depressed individuals to the antidepressant fluoxetine found that nonresponders performed significantly worse on pre-treatment measures of EF (Wisconsin Card Sorting Task, Stroop task; Dunkin et al., 2000). Determining which deficits come first, or understanding the causal and temporal mechanisms of the relationship between difficulties in EF and psychopathology, will depend in part on the availability of longitudinal data. It is likely that the relationships among EF, emotion, and motivation are bidirectional and/or multidirectional, such that deficits in one foster deficits in another, creating a snowball effect and in turn, exacerbating the initial deficits.

Regardless of the nature of causality among these psychological and biological processes (Miller, 2010), the relationships among EFs, emotion, and motivation in anxiety and depression are likely related to dysfunction in brain networks that are involved in integrating aspects of these processes, particularly DLPFC and ACC (Gray et al., 2002; Koechlin and Hyafil, 2007; Kouneiher et al., 2009; Pessoa, 2009). Evidence suggests that DLPFC and ACC merge input from various regions involved in subprocesses of cognition, emotion, and motivation (Gray et al., 2002; Gray, 2004; Pessoa, 2008, 2009; Spielberg et al., 2012a,b). DLPFC has substantial connectivity to regions involved in determining the emotional significance and motivational value of stimuli, including more medial PFC structures, such as pre-supplementary motor area (pre-SMA), dACC (Kouneiher et al., 2009), and frontopolar cortex (Koechlin and Hyafil, 2007). Further, research in our laboratory showed that DLPFC regions associated with approach and avoidance motivation demonstrated increased connectivity with OFC, ACC, amygdala, and basal ganglia during an EF task involving goal maintenance in the face of distraction (Spielberg et al., 2012a).

ACC also seems a likely candidate for integrating aspects of emotion, motivation, and EF, evidenced by its connectivity to both the amygdala and nucleus accumbens, as well as OFC and ventral striatum (Pessoa, 2009), key areas involved in emotion and motivation. Hence, subregions of ACC are involved in assessing events for their emotional and motivational relevance, error and conflict monitoring, and predicting value of potential rewards and punishments (Rushworth et al., 2004, 2007; Banich, 2009; Pessoa, 2009). In addition, DLPFC and ACC interact in order to utilize emotional and motivational information to develop and implement goal-directed strategies (Beckmann et al., 2009; Spielberg et al., 2012a). In anxiety and depression, DLPFC and ACC appear to be dysfunctional in integrating emotion- and motivated-related information when recruited to implement cognitive control/EFs and exhibit decreased connectivity (Silton et al., 2011).

Other research explicitly examining functional connectivity between regions also suggests that anxiety and depression are associated with dysfunctional communication between regions. For example, individuals with MDD exhibited decreased connectivity in a fronto-parietal network relative to healthy controls during a working memory task (Vasic et al., 2009). Individuals with social phobia displayed less functional connectivity between the amygdala, medial OFC, and PCC than healthy individuals during rest (Hahn et al., 2011), as well as altered connectivity between various regions (e.g., amygdala, mPFC, inferior parietal lobule) during a face perception task (Danti et al., 2010). Thus, it is likely that the dysfunction observed in individuals with anxiety and depression is related to problematic communication between regions, rather than just altered activity in isolated regions.

## Intervention and neuroplasticity

Numerous interventions, both psychological and biological, have been developed to target disruptions in cognition, emotion, and motivation interactions associated with anxiety and depression. In addition, a growing body of research has aimed to elucidate the mechanisms of neuroplasticity by characterizing the experience-dependent functional and structural changes in the brain associated with these interventions. As reviewed above, anxiety and depression are associated with impaired executive control, dysfunctional relationships among cognitive, emotional, and motivational processes, and abnormal activity in brain regions that are part of networks implementing these processes. Psychological/behavioral, pharmacological, and direct physiological (e.g., electroconvulsive therapy [ECT]) interventions have been shown to reduce emotional symptoms, decrease negative thoughts and beliefs, and alter maladaptive motivational and behavioral styles (for reviews, see Mayberg, 2000; Mayberg et al., 2005; DeRubeis et al., 2008; Frewen et al., 2008; Clark and Beck, 2010). Importantly, they appear to normalize function and structure in the brain regions and networks that exhibit dysfunction prior to treatment in individuals who respond to treatment.

Although a large body of literature demonstrates improvements in psychological symptoms associated with various types of interventions, it should be noted that not everyone responds to one or more of these treatments. For example, Cognitive Therapy (CT), a type of psychotherapy with much empirical support, is effective for approximately 40-60% of individuals with depression (APA, 2000). Less than half of individuals with depression who receive either psychotherapy or pharmacotherapy are able to attain full remission (Casacalenda et al., 2002). Butler and colleagues (1991) found that only 32% of individuals with Generalized Anxiety Disorder (GAD) who received CT scored within the healthy range on three measures of anxiety immediately after treatment. Further, many individuals who respond initially to treatment ultimately relapse, regardless of the type of treatment received. However, there is evidence that psychotherapy leads to lower relapse rates than does pharmacotherapy (Gould et al., 1995, 1997; Hollon et al., 2006). There continues to be great room for improvement in treatments in order to increase recovery rates and prevent relapse. If we can better understand the psychological and neural mechanisms through which treatment is effective for responders, this knowledge can be used to improve treatments, as well as match specific treatments to those who are likely to benefit from it.

Cognitive behavioral therapy (CBT) is one of the most effective psychological treatments for anxiety and depression and addresses emotion-cognition and motivation-cognition interactions that are altered in these disorders. The cognitive component of CBT (and CT) emphasizes changing problematic patterns of thinking and maladaptive beliefs, which leads to improvements in emotional and motivational function and enhances approach behavior. The behavioral component of CBT and a related therapeutic approach, Behavioral Activation (BA), target problematic behavioral patterns (e.g., avoidance of negative stimuli/situations and punishment-related outcomes) and use positive reinforcement to facilitate engagement in pleasant, rewarding activities (Martell et al., 2001; Kuyken et al., 2005). In addition to increasing more adaptive, approach-related behaviors, these behavioral strategies lead to alterations in cognition and emotion. Thus, CBT and CT emphasize the interconnection of thoughts, emotions, and motivations.

Successful CBT/CT for anxiety and depression has consistently been shown to alter activity in several brain regions, including DLPFC, VLPFC, and ACC (for reviews, see Frewen et al., 2008; Clark and Beck, 2010; Miller, 2010). Some studies have found that CBT and CT for depression are associated with decreased amygdala activation and increased prefrontal activation during tasks that recruit various cognitive, emotional, and motivational processes relative to pre-treatment activation (see DeRubeis et al., 2008). Others have found that prefrontal activation decreased during a resting-state condition (e.g., Goldapple et al., 2004). It has been suggested that maintaining lower frontal resting-state activity is adaptive in that it allows for more flexible activity during EF task conditions, with the amount of activity depending on the context and task demands (DeRubeis et al., 2008).

Similar to CBT studies of depression, studies of successful CBT for anxiety disorders highlight the neuroplasticity of several brain regions that play key roles in cognition, emotion, and motivation. For example, individuals diagnosed with posttraumatic stress disorder (PTSD) were given treatment involving imaginal exposure to feared situations and cognitive restructuring, two key components of CBT for PTSD that target avoidance behaviors and distortions in thought patterns (Felmingham et al., 2007). Researchers found that treatment was associated with PTSD-symptom improvement, as well as increased rACC activation and decreased amygdala activation when viewing fearful versus neural faces. Thus, treatment normalized dysfunctional pre-treatment activity in regions involved in emotional experience and regulation.

Treatment for obsessive-compulsive disorder (OCD) that focused on changing maladaptive behavior patterns was associated with decreased caudate activity during rest (Schwartz et al., 1996; Nakatani et al., 2003) as well as alterations in functional connectivity between areas in the caudate-orbital-thalamic circuit for CBT treatment responders (Baxter et al., 1992; Schwartz et al., 1996). Individuals with spider phobia exhibited decreased symptoms post-CBT along with significant reductions of pre-treatment hyperactivity in insula and ACC (Straube et al., 2006) as well as DLPFC and parahippocampal gyrus (Paquette et al., 2003). Clark and Beck (2010) reviewed studies of CBT for anxiety disorders and reported that treatment leads to increased activity in ventral and dorsal ACC, mPFC, and VLPFC, regions that exhibit pre-treatment hypoactivity relative to controls, as well as decreased activity in amygdala, hippocampus, and anterior and medial temporal cortex, which show pre-treatment hyperactivity. Thus, CBT alters activity in regions involved in diverse cognitive, emotional, and motivational processes, including more bottom-up, stimulus-driven processing and top-down processing (Clark and Beck, 2010).

As with CBT/CT, numerous studies examining the effects of antidepressant medication treatment have found decreases in depressive symptoms with concomitant alterations in activation in several brain regions involved in a range of cognitive, emotional, and motivational processes. Successful antidepressant treatment has been associated with decreased activation in regions involved in threat and punishment-related responses such as the amygdala, subgenual cingulate, and striatum in response to affective stimuli (Mayberg et al., 2000; Sheline et al., 2001; Davidson et al., 2003; Fu et al., 2004). Prior to treatment, these regions appeared to be hyperactive relative to healthy individuals. In addition, antidepressant treatment has been shown to increase activation in several cognitive control regions that are typically hypoactive in depressed individuals, including prefrontal cortex and rACC (Mayberg et al., 2000; Davidson et al., 2003; Fu et al., 2004). Supporting these results, a meta-analysis of 9 studies found that antidepressant treatment for depression was associated with increased activation in DLPFC, VLPFC, and dorsomedial PFC, along with decreased activation in amygdala, hippocampus, parahippocampal gyrus, ACC, PCC, OFC, insula, and parietal regions (Delaveau et al., 2011). It has been proposed that antidepressant medication does not target prefrontal activity directly; rather, it targets amygdala activity, which in turn prompts prefrontal disinhibition (DeRubeis et al., 2008) with the effect of increasing activity supporting cognitive control. Further, antidepressant medication appears to enhance functional connectivity among brain regions in depressed individuals (Anand et al., 2007), shown in other work to be disrupted (e.g., Silton et al., 2011).

Antidepressant medication has been used to treat anxiety as well. Studies examining its effects on neural activity in individuals with anxiety disorders have found that it also appears to normalize activity in regions and networks that were dysfunctional prior to treatment in medication responders. For example, obsessive-compulsive disorder (OCD) has been associated with hyperactivity in frontal-subcortical circuits relative to healthy individuals, and antidepressant treatment has been shown to decrease activity in OFC and caudate nucleus (Saxena et al., 1999). In addition, antidepressant treatment has been associated with decreased activity in medial temporal cortex in individuals with PTSD (Seedat et al., 2004). Further, after antidepressant treatment, individuals with social phobia displayed attenuated activity in amygdala, hippocampus, and parahippocampal cortex during a public speaking task (Furmark et al., 2002, 2005).

These functional changes associated with successful medication and psychotherapy treatment are supported by structural changes. Antidepressants appear to reverse various structural abnormalities observed in depression and anxiety. For example, there is evidence that chronic antidepressant treatment enhances neurogenesis, prevents neuronal atrophy, and promotes neuronal sprouting and dendritic branching (Vaidya and Duman, 2001; Pittenger and Duman, 2008). It also stimulates new synapse formation, strengthens synaptic connectivity, and alters neurotrophic signaling cascades (Manji et al., 2003; Pittenger and Duman, 2008; Andrade and Rao, 2010). These cellular and molecular changes are associated with more macro-level changes, including increased regional brain volume (e.g., hippocampus; Vermetten et al., 2003; Malykhin et al., 2010). There is little direct evidence of cellular and regional changes associated with psychotherapy specifically; however, such changes have been observed after various learning-related experiences similar to those involved in psychotherapy (Kolb and Whishaw, 1998; Liggan and Kay, 1999), and the neuroplastic effects of structured behavioral interventions more generally are well established (e.g., Elbert et al., 1995).

Studies examining the neurobiological effects of pharmacological versus psychological treatments have been inconsistent, with some reporting similar results (e.g., Baxter et al., 1992; Furmark et al., 2002), and others reporting diverging results (for reviews, see Mayberg, 2003; DeRubeis et al., 2008). Seminowicz and colleagues (2004) asserted that different types of treatment (e.g., CBT, medication) alter activity in some of the same regions, though in different ways (e.g., CBT increases or decreases activity in a region, whereas medication does the opposite). Regardless, psychotherapy and antidepressant medication appear to have at least some similar effects, though they likely have distinct mechanisms of change (e.g., Kumari, 2006; DeRubeis et al., 2008). It has been hypothesized that CBT/CT and antidepressant medication both ultimately affect prefrontal, limbic, and subcortical regions, though they differ in their “proximal mechanisms of action” and direct targets, such that CBT/CT directly enhances prefrontal function and top-down emotion regulation and cognitive control, whereas antidepressant medication alters amygdala activation and bottom-up, stimulus-driven processes (Linden, 2006; DeRubeis et al., 2008). This hypothesis is consistent with anecdotal reports that medication can be helpful in diminishing the intensity of emotional and motivational symptoms in a way that allows more intentional cognitive strategies to be deployed effectively. This may explain why the combination of antidepressants and CBT is more effective than either alone in difficult-to-treat cases of depression (Keller et al., 2000b). To our knowledge, no research has examined the neural changes associated with combined medication and psychotherapy treatment. Future research in this area will be useful to determine if pharmacotherapy and psychotherapy have additive or interactive effects on brain activation.

The studies reviewed above are limited in that they reflect neural changes in individuals who responded to treatment and showed at least some symptom improvement. However, as mentioned above, numerous individuals do not respond to medication and/or psychotherapy. Although uncommon, a few studies have examined neural patterns in treatment nonresponders. For example, Mayberg and colleagues (2000) found that, relative to responders, nonresponders showed an inverse activation pattern in some areas (e.g., hippocampus, PCC) as well as no change in subgenual cingulate and prefrontal cortex. In addition, an exciting line of research has begun to examine how findings from studies of neural mechanisms involved in psychological and pharmacological interventions can be used to inform treatment selection for individuals, given that not everyone responds. Numerous studies have found that pre-treatment activity in rACC and subgenual portions of ACC (sgACC) is consistently predictive of who will respond to treatment (for a review, see Mayberg, 2003). For example, Siegle and colleagues (2006) scanned depressed individuals prior to 16 sessions of CBT while they performed an emotional information processing task. They found that low pre-treatment sgACC and high amygdala activation in response to negative words were associated with increased response to CBT. The results regarding sgACC were replicated in two separate samples (Siegle et al., 2012), suggesting that baseline sgACC activity is a reliable measure that can be used to increase response rates by providing CBT to those individuals most likely to benefit from it. Such evidence of pretreatment psychophysiological reactivity predicting psychotherapy response adds to a long tradition of such literature (e.g., Lang et al., 1970).

Similar to CBT, several antidepressant studies have found that greater pre-treatment activity in rACC consistently predicted better response to antidepressant treatment in individuals with anxiety and depressive disorders (Mayberg et al., 1997; Pizzagalli et al., 2001; Davidson et al., 2003; Whalen et al., 2008; Nitschke et al., 2009). Activity in other regions, including OFC and amygdala, has also been found to predict greater improvement with treatment (Saxena et al., 1999; McClure et al., 2007). In addition, patterns of connectivity between regions in a network shown to be dysfunctional in depression (e.g., PFC, sgACC, OFC, hippocampus) have been used to distinguish antidepressant medication responders from nonresponders (Seminowicz et al., 2004). Measures of pre-treatment structural neuroanatomy, particularly rACC volume, have also been used to predict response to antidepressant medication and CBT in individuals with MDD and PTSD, respectively (Bryant et al., 2008; Costafreda et al., 2009). Although much work remains to be done before routinely applying such findings to clinical practice, matching individuals with treatments likely to be effective based on pretreatment psychophysiological and neuroantatomical characterization is a promising method that can be used in the future to enhance response rates.

## Cognitive bias modification

Another line of research has explored improving response rates with strategies other than treatment-matching. Some researchers have argued that using methods that more directly target cognitive processes, specifically the biases observed in anxiety and depression, will improve current treatment approaches. Thus far, evidence suggests that decreasing cognitive biases leads to enhanced emotional function (for review, see Koster et al., 2009; Hertel and Mathews, 2011). This literature developed in part to test the theory that cognitive biases play a role in the etiology of anxiety and depressive disorders and are an important target for therapeutic intervention. Numerous studies have now provided support that cognitive biases 1) play a causal role in psychopathology, 2) can be modified, and 3) lead to improvements in clinical symptoms and emotional reactivity to stress when these biases are reduced or alleviated. In fact, cognitive bias modification (CBM) has received so much recent attention that a special section in *Journal of Abnormal Psychology* (Volume 118, Number 1) was devoted to it, numerous reviews have already been published (e.g., Beard, 2011; Hertel and Mathews, 2011; MacLeod, 2012), and meta-analyses have been conducted (e.g., Hakamata et al., 2010; Hallion and Ruscio, 2011; Beard et al., 2012). This literature encompasses a variety of experimental procedures, typically computerized, that are used to systematically alter cognitive processing styles.

Given the prolific research focusing on the role of attentional bias in anxiety, it is not surprising that there is also a large CBM literature investigating the alteration of this bias (for review, see Bar-Haim, 2010). For example, individuals with GAD exhibited reduced anxiety symptoms after undergoing a training procedure involving a probe task that induced a bias to orient attention away from threatening information toward neutral words (Amir et al., 2009). In fact, 50% of those individuals in the 8-session computer training condition no longer met criteria for a diagnosis of GAD after training versus 13% in the control condition. These results provide support for the assertion that an attentional bias to negative information plays a causal role in the development of GAD symptoms. Similarly, individuals who suffered from recurrent depression exhibited significant reductions in depression, anxiety, automatic negative thoughts, and rumination after undergoing attention training involving monitoring external auditory stimuli under conditions of selective attention, attention switching, and divided attention (Papageorgiou and Wells, 2000).

Research has also found that modifying attentional biases buffers against the negative effects of stressors in real-world contexts (Hakamata et al., 2010). For example, See and colleagues (2009) found that, in addition to reducing trait anxiety scores, an attentional bias modification procedure led to decreased state anxiety in response to the real-life stress associated with moving to a new country to start college. In a series of studies, Dandeneau and colleagues (2007) demonstrated that attentional training reduced a bias toward threatening social information and led to decreased stress responses in both school and work settings. Based on their meta-analysis, Hallion and Ruscio (2011) proposed that cognitive biases exert their influence on anxiety and depressive symptoms only after being activated by stressors.

In addition to targeting attention, cognitive bias modification procedures have also been developed to alter other types of biases, including the negative interpretation bias observed in anxious individuals and the overgeneral autobiographical memory bias that accompanies depression. Hirsch and colleagues (2009) implemented a procedure that allowed individuals high in worry to practice accessing benign instead of threatening meanings of homographs and emotionally ambiguous scenarios. These individuals reported fewer negative thought intrusions and less worry during a breathing focus task than participants in a control training condition. Further, individuals who underwent the benign-meaning training demonstrated greater residual working memory capacity despite being instructed to worry, suggesting that this intervention also enhances a key cognitive process that appears to play a role in anxiety development and exacerbation.

Several studies have demonstrated that interpretation biases contribute to observed distortions in memory (for review, see Hertel and Brozovich, 2010; Hertel and Mathews, 2011). Thus, alleviating this bias likely improves memory as well. As reviewed above, depression is associated with overgeneral autobiographical memory. Watkins and colleagues (2009) found that providing concreteness training to dysphoric individuals reduced their tendency to engage in abstract and overgeneral processing and decreased depressive symptoms, rumination, and self-criticism.

Little is known about the neural mechanisms associated with the psychological changes induced by CBM procedures. One study examining the effects of attentional training with healthy individuals found altered activity in lateral PFC spanning dorsolateral and ventrolateral regions during a novel attention task (Browning et al., 2010). Specifically, activity in lateral PFC increased when participants attended to faces that were the valence they were trained to avoid (i.e., fearful faces for those in the avoid-threat condition, neutral faces for those in the attend-threat condition). In addition, connectivity analyses indicated that lateral PFC influenced activity in visual sensory cortex, consistent with studies showing that both regions are part of a network involved in control of attention.

It has been suggested that the mechanisms through which CBM procedures exert their effects are distinct from those associated with CBT and pharmacological interventions, specifically that they operate at different stages of processing (e.g., Browning et al., 2010). However, conflicting theories exist about which stage of processing CBM affects. For example, Browning and colleagues (2010) suggested that, whereas pharmacological interventions affect the initial deployment of attention and involve a bottom-up, stimulus-driven system including the amygdala, CBM targets later stages of attentional processes involving PFC. In contrast, Hallion and Ruscio (2011) asserted that CBM targets earlier, more automatic cognitive processes, whereas CBT targets later stages. Future work will likely benefit from employing hemodynamic and electromagnetic neuroimaging methods to help determine which stages of processing are affected by various interventions.

There are numerous additional questions to address regarding CBM. For example, it is not clear how much training is needed (e.g., number and length of sessions), how long their effects last, how effective CBM techniques are relative to other treatments, or what factors moderate their effectiveness. The CBM literature has not explicitly considered the impact that such interventions may have on motivational processes, including real-life behavioral outcomes. However, it is likely that CBM-induced improvements in cognitive and emotional function translate into enhanced motivational function, such as decreasing avoidance and increasing pro-social behavior. Future work will need to test this hypothesis. In addition, several researchers have suggested that combining CBM with other therapies (e.g., CBT) may enhance their effectiveness (e.g., Bar-Haim, 2010; Browning et al., 2010; Hallion and Ruscio, 2011), but this has yet to be systematically assessed. To the extent that EF deficits actually drive biases associated with anxiety and depression, it may be that CBM procedures actually enhance EF processes (e.g., control of attention, working memory capacity) that in turn reduce biases. Thus, using interventions that more directly target specific EFs (e.g., inhibition, working memory) may be even more effective and lead to more long-lasting changes.

## Executive function training

Evidence is accruing that EF can improve with training (e.g., Olesen et al., 2004; Erickson et al., 2007; Dahlin et al., 2008) and that interventions targeting specific EFs directly are associated with improvements in symptoms of psychopathology (e.g., Papageorgiou and Wells, 2000; Siegle et al., 2007). A small but growing number of studies demonstrate that training-related increases in working memory ability can yield improvements in a range of cognitive skills (Chein and Morrison, 2010; Jaeggi et al., 2011; Brehmer et al., 2012), improvements in cognitive function in clinical populations with known inhibitory impairment (e.g., Klingberg et al., 2005; Popov et al., 2011), and improvements in quality of life (e.g., Vogt et al., 2009). The generalizability of training-related increases in working memory ability to non-trained tasks is hypothesized to occur when the transfer task recruits overlapping cortical regions (e.g., Jonides, 2004; Olesen et al., 2004). Identifying specific EF deficits and their associated neural mechanisms in anxiety and depression could improve the focus of cognitive remediation interventions, as well as their transfer effects to real-world applications (e.g., promoting goal attainment, approach behavior, or emotion-regulation abilities).

Because of the importance of working memory in general cognition (Kane and Engle, 2002), many cognitive training programs have been developed to target it. The hope has been that related cognitive abilities (e.g., inhibition, updating, attention) will subsequently improve and lead to enhanced emotional and motivational function. Numerous studies have shown that frontal and parietal regions are key nodes in a network involved in implementing working memory (for a review, see D'Esposito, 2001). Working memory training with healthy individuals was associated with increases in activation in prefrontal and parietal regions, specifically middle frontal gyrus and superior, intraparietal and inferior parietal cortex (Olesen et al., 2004). Not surprisingly, research suggests that working memory capacity is correlated with the structural integrity of white matter connecting frontoparietal regions (Klingberg, 2006). Working memory training increased the white matter structural integrity of a region adjacent to intraparietal sulcus, which connects this region to frontal cortex, and a region adjacent to the body of the corpus collosum, which connects bilateral DLPFCs (Takeuchi et al., 2010). It was hypothesized that more effective communication of brain regions via increased myelination accounts for enhanced working memory post-training.

As interest in the potential role of EF as a target of intervention is increasing (Chein and Morrison, 2010; Jaeggi et al., 2011; Brehmer et al., 2012), identification of specific EF deficits and associated patterns of brain activity in psychopathology will likely serve the development and/or modification of effective interventions (such as “neurobehavioral interventions” as discussed in Siegle et al., 2007). In fact, difficulties with different aspects of EF may present barriers to current treatment methods. For example, an individual who has trouble shifting might need help planning strategies to transition more easily between daily tasks. It has been shown that the efficacy of current psychological treatments depends on adequate EF (Mohlman and Gorman, 2005). For example, CBT involves reappraisal, hypothesis generation, and self-monitoring, which all require EF (Mohlman and Gorman, 2005; Gotlib and Joormann, 2010). In addition, there is some evidence that EF training actually improves response to CBT (Mohlman, 2008), although research is needed to examine which aspects of EF are most crucial for the efficacy of these interventions and might benefit most from training. More research is clearly needed to explore how EF training might improve treatment outcomes.

Many individuals do not fully recover after receiving therapy or relapse after therapy has completed (Kendall and Sugarman, 1997; DeRubeis et al., 1999). It may be advantageous for these individuals to receive interventions that initially target and enhance EFs, which could allow them to engage in and benefit more from other components of the treatment. There is some preliminary data that adding EF training to treatment as usual (TAU) leads to better outcomes in depressed individuals (Siegle et al., 2007). Specifically, Siegle and colleagues added Cognitive Control Training (CCT) to enhance working memory and attention to TAU, which included group psychotherapy, case management, and psychotropic medication. They found that individuals who received CCT in addition to TAU displayed greater improvements in depressive symptoms than did those in the TAU alone condition, as well as normalization of activation in DLPFC and amygdala.

## Mindfulness-based intervention

Not much research has been conducted examining the outcomes of specific EF training procedures utilizing EF tasks in individuals with anxiety and depression beyond the preliminary study described above. However, outcomes related to mindfulness are an area of increasing interest because it is considered an intervention that trains control of attention and other EFs. A large body of evidence has demonstrated that mindfulness-based stress reduction (MBSR) is an effective intervention for a range of psychological disorders, including anxiety and depression (Hofmann et al., 2010). It has been hypothesized that the improvement in emotional symptoms associated with mindfulness is due to the fact that it utilizes cognitive strategies that involve strengthening EFs, including sustaining attention, flexibly switching the focus of attention, and inhibiting elaborative processing (Bishop et al., 2004). Mindfulness interventions have been associated with significant improvements in performance on working memory and sustained attention tasks, as well as concomitant decreases in rumination, depressive symptoms, and negative affect relative to a control group (Chambers et al., 2008). Mindfulness also appears to decrease rates of relapse in individuals who have experienced several depressive episodes (Teasdale et al., 2000). Similar to CBM, the mindfulness literature has not directly assessed alterations in motivation-related processes and behaviors, though it is likely that the improvements in cognitive and emotional function enhance motivational processing.

The cognitive improvements and symptom reductions gained through mindfulness training are accompanied by mindfulness-induced neuroplasticity (for a review, see Holzel et al., 2011). Healthy adults who completed an 8-week MBSR training course and expert meditators exhibited reduced activation in brain areas associated with a visceral sense of self, including anterior insula, ventral ACC, and mPFC during the act of meditation (Ives-Deliperi et al., 2011) and while processing emotional stimuli, which also corresponded with reduced amygdala activation (Desbordes et al., 2012). These effects suggest that experience with MBSR and other types of meditation results in reduced reactivity to both physical and emotional stimuli. In addition, there is evidence of increases in activity in brain regions associated with attention and executive control. Studies have observed increases in PCC during active meditation (Ives-Deliperi et al., 2011) and less activity in major nodes of the default-mode network, including mPFC and PCC, during periods of rest in experienced meditators (Brewer et al., 2011), suggesting decreased mind wandering. A study utilizing ERPs during a Stroop task found that individuals with MBSR experience displayed increased early-latency responses recorded over right posterior cortex to all stimuli, suggesting increased deployment of early attentional resources, and reduced later centro-parietal potentials to all stimuli but especially incongruent stimuli, indicating more efficient processing and control of these conflict stimuli (Moore et al., 2012). Although little research has examined these effects in clinical populations, one study found that individuals diagnosed with social anxiety disorder (SAD) who underwent MBSR training exhibited reduced amygdala activation and increased dorsomedial PFC, ventromedial PFC, mPFC, and PCC activation in response to negative stimuli (Goldin and Gross, 2010). These effects suggest that these individuals were better able to control their emotional response to negative stimuli via reduced bottom-up, stimulus-driven reactivity and/or increased top-down control.

Other noteworthy neuroimaging effects observed in individuals with MBSR experience comes from research employing techniques that examine structural changes in the brain. Increased cortical thickness has been observed in several regions in individuals with MBSR experience, including PFC, PCC, OFC, hippocampus, and anterior insula (Lazar et al., 2005; Luders et al., 2009; Holzel et al., 2011). These increases in gray matter density were found to positively correlate with meditation experience (Lazar et al., 2005). Individuals with MBSR experience have been shown to exhibit increased connectivity among major fiber tracts in the brain, including whole brain fiber tracts, major tracts in both hemispheres, and the two largest interhemispheric fiber tracts than did healthy controls (Luders et al., 2011). Brewer et al. (2011) found increased connectivity among DLPFC, dACC, and PCC in experienced meditators, which again suggests increased self-monitoring ability and enhanced cognitive control. Finally, increased gyrification, or an increase in cortical gray matter and synaptogenesis, has been observed in precentral gyrus, fusiform gyrus, cuneus, and dorsal insula in individuals with MBSR experience (Luders et al., 2012). Among these areas showing increased gyrification, only dorsal anterior insula was correlated with meditation experience. This area is involved in integrating aspects of autonomic, affective, and cognitive processes and may contribute to decreased mind wandering, daydreaming, and ruminating, which are all key aspects of successful meditation.

Although the study by Goldin and Gross (2010) appears to be the only one to directly examine the neural effects of MSBR in individuals diagnosed with an anxiety or depressive disorder thus far, the growing body of research on brain changes associated with MBSR in healthy populations has implications for how it may mitigate or prevent anxiety or depression. Some of the neuroplastic effects observed in healthy individuals with MBSR experience occur in areas exhibiting dysfunction in anxiety and depression, as reviewed above. Thus, it is likely that MBSR practice in individuals with anxiety and depression normalizes activity in these regions, in addition to reducing symptoms and increasing control over rumination and worry.

## Limitations and future directions

In terms of neuroplasticity, many of the structural changes have been examined in relation to medication. Much less work has been done to understand the structural changes associated with psychological interventions. In fact, a pervasive premise, and not only among the general public, is that biological abnormalities should be treated with biological interventions. Yet there is now abundant evidence that psychological treatments alter biology, just as biological treatments alter psychology (Miller, 1996, 2010). Further, despite a large body of research examining the functional changes associated with various types of psychological and biological interventions, there is much we do not yet know because of the limited contexts in which these changes have been assessed. These functional changes have been assessed almost entirely using tasks tapping basic emotional processing (e.g., viewing negative versus neutral faces). Understanding of the neural changes associated with such interventions would be greatly enhanced by examining changes across a variety of tasks and contexts recruiting a range of cognitive, emotional, and motivational processes. This would permit testing whether interventions lead to greater flexibility and dynamic range of neural activity, such that the degree of activation depends on the context and task demands rather than being habitually high or low, or whether interventions lead to consistently moderate responses.

In addition to research that examines a broader range of contexts, interventions would greatly benefit from future work that is informed by the psychological and biological research reviewed in the present paper. Current treatments (e.g., CBT, medication) may be enhanced by the initial implementation of targeted strategies that more directly boost activity in EF-related regions (e.g., cognitive control/working memory training) and/or decrease activity regions that play key roles in initial reactivity to stimuli (e.g., mindfulness). Although these strategies may not be sufficient alone, they could potentially address specific deficits that in turn allow individuals to more fully engage in challenging treatment techniques. Further, research on shared brain mechanisms that contribute to various forms of psychopathology (e.g., connectivity between DLPFC and ACC) could inform nonspecific treatment strategies that address symptoms present in a range of disorders (Siegle et al., 2007).

Several other methodological and theoretical limitations that are pervasive in the field also need to be addressed. The vast majority of the treatment studies reviewed reported results at the level of individual areas. However, the field is moving towards a network approach in order to better understand interactions among cognitive, emotional, and motivational processes, which involve a complex array of operations that engage distributed networks of brain regions. There is some, albeit minimal evidence starting to accrue that treatment normalizes functional communication between regions in individuals with anxiety and depression. Anand et al. (2005) found that after 6 weeks of antidepressant treatment, individuals with depression exhibited increased connectivity between ACC and various regions (amygdala, thalamus, and striatum) at rest and after viewing neutral and positive but not negative pictures. Further, measures of pre-treatment connectivity, rather than just the activity of a single region, may also be useful for predicting who will respond to treatment. Salvadore and colleagues (2010) found that less functional connectivity between pregenual/subgenual ACC and left amygdala during a working memory task prior to antidepressant treatment was associated with greater symptom improvement post-treatment. Thus, the field would greatly benefit from future studies that utilize a network perspective in order to better understand the mechanisms through which various treatments exert their effects.

As reviewed above, various antidepressant and psychological treatments appear to target processes that rely heavily on top-down EF (e.g., interpretation of negative information), as well as dampen reactivity to emotional stimuli. This is reflected in treatment-related enhancements of activity in regions involved more in top-down processing and decrements in activity in regions involved more in bottom-up, stimulus-driven processing. Although some researchers have theorized that a specific type of treatment primarily targets one or the other type of processing, it is likely that ultimately both are affected, given the functional connectivity and interactive nature of the systems/networks that implement these processes. Future work explicitly examining functional connectivity should directly test this hypothesis.

In addition, very few studies take into account the frequency at which anxiety and depression co-occur (Sanderson et al., 1990; Kessler et al., 1994; Brown et al., 2001). Comorbidity is present in at least one-half of those diagnosed with an anxiety or depressive disorder (for reviews, see Gersh and Fowles, 1979; Breier et al., 1985; Clark, 1989) and leads to a greater impact than either disorder alone. Comorbidity is associated with greater impairments in psychosocial function, greater severity of disorder, elevated rates of suicidality and morbidity, increased health service use, increased treatment resistance, and poorer short- and long-term outcomes (Judd et al., 1998; Lydiard and Brawman-Mintzer, 1998). Without taking comormidity into account, it is unclear whether patterns of brain activity are specific to depression or anxiety or if instead they reflect their co-occurrence. Some evidence indicates that co-occurring anxiety and depression have additive and interactive effects on brain function (e.g., Bruder et al., 1997; Keller et al., 2000a; Kentgen et al., 2000; Pizzagalli et al., 2002; Engels et al., 2010). Much work needs to be done to better understand how co-occurring levels of anxiety and depression alter brain network function during tasks involving a range of cognitive, emotional, and motivational processes as well as how treatment alters these patterns.

Another issue that warrants consideration in the hemodynamic neuroimaging treatment literature is the reliability of the blood-oxygen-dependant-level (BOLD) signal across time, given that various factors that can affect it (e.g., caffeine, nicotine, movement, breathing rate; MacDonald and Jones, 2009). Carrig and colleagues (2009) reviewed research investigating the test-retest reliability of fMRI and determined that studies examining intraclass correlation coefficients (ICC) have found good to excellent reliabilities. However, Plichta and colleagues (2012) found that the stability of within-subject amplitude varied depending on the specific task being examined (emotional vs. motivational and cognitive). Little work has been done in examining the reliability of the BOLD signal specifically in patients, an issue that is particularly relevant for the treatment literature. One study found that individuals with schizophrenia exhibited low reliability (ICC = 0.2) in DLPFC, whereas control participants exhibited excellent reliability (ICC = 0.81; Manoach et al., 2001). Thus, future research would benefit from examining reliability of the BOLD signal in individuals with anxiety and depression prior to treatment.

## Conclusion

It has become clear just how interconnected the cognitive, emotional, and motivational deficits in anxiety and depression are, such that it is difficult to distinguish their influences. The present review has demonstrated how basic research on the relationships among cognition, emotion, and motivation in psychopathology and related neural mechanisms has been used to inform treatment-related research. In fact, there continues to be rich potential for the synergy between these literatures. Despite numerous advances, we do not fully understand the mechanisms that lead to psychopathology, or how to harness these mechanisms most effectively for successful interventions.

This review has highlighted numerous gaps in the literature. It is clear that motivation is related to the cognitive and emotional symptoms observed in psychopathology, but little work has been done to understand exactly how motivation interacts with and affects emotion and cognition. Additionally, much of the treatment-related research has focused on emotion-cognition interactions and neglected to examine how interventions may lead to alterations in motivational processes. This work could lead to the development and refinement of treatments that better target the motivational deficits observed in psychopathology. Further, there is much excitement about the application of CBM procedures and EF training to better treat psychopathology, but much research remains to be done before these methods are used in common practice. For example, it is not clear how their effects translate to everyday performance or how long they last. If it is determined that they are as effective as current treatment methods or useful in improving the effectiveness of current methods, these training paradigms could likely be employed easily at home, via internet or computer software, for little cost. There is much promise in capitalizing on the synergy between neuroscience and intervention research to better prevent and treat psychological disorders.

### Conflict of interest statement

The authors declare that the research was conducted in the absence of any commercial or financial relationships that could be construed as a potential conflict of interest.

## References

[B1] Airaksinen E. Larsson M. Forsell Y. (2005). Neuropsychological functions in anxiety disorders in population-based samples: evidence of episodic memory dysfunction. J. Psychiatr. Res. 39, 207–214 10.1016/j.jpsychires.2004.06.001 15589570

[B2] Alexopoulos G. S. Meyers B. S. Young R. C. Kalayam B. Kakuma T. Gabrielle M. (2000). Executive dysfunction and long-term outcomes of geriatric depression. Arch. Gen. Psychiatry 57, 285–290 10.1001/archpsyc.57.3.285 10711915

[B3] Amir N. Beard C. Burns M. Bomyea J. (2009). Attention modification program in individuals with generalized anxiety disorder. J. Abnorm. Psychol. 118, 28–33 10.1037/a0012589 19222311 PMC2645540

[B4] Anand A. Li Y. Wang Y. Gardner K. Lowe M. (2007). Reciprocal effects of antidepressant treatment on activity and connectivity of the mood regulating circuit: an fMRI study. J. Neuropsychiatry Clin. Neurosci. 19, 274–282 10.1176/appi.neuropsych.19.3.274 17827412 PMC3465666

[B4a] Anand A. Li Y. Wang Y. Wu J. Gao S. Bukhari L. (2005). Antidepressant effect on connectivity of the mood-regulating circuit: an fMRI study. Neuropsychopharmacology 30, 1334–1344 10.1038/sj.npp.1300725 15856081

[B5] Andrade C. Rao N. S. K. (2010). How antidepressant drugs act: a primer on neuroplasticity as the eventual mediator of antidepressant efficacy. Indian J. Psychiatry 52, 378–386 10.4103/0019-5545.74318 21267376 PMC3025168

[B6] APA. (2000). Practice guideline for the treatment of patients with major depressive disorder (revision). Am. J. Psychiatry 157, 1–45 10.1176/appi.pn.2013.5a13 10767867

[B7] Austin M. P. Mitchell P. Goodwin G. M. (2001). Cognitive deficits in depression. Br. J. Psychiatry 178, 200–206 10.1192/bjp.178.3.200 11230029

[B8] Banich M. T. Milham M. P. Atchley R. A. Cohen N. J. Webb A. Wszalek T. (2000). fMRI studies of Stroop tasks reveal unique roles of anterior and posterior brain systems in attentional selection. J. Cogn. Neurosci. 12, 988–1000 10.1162/08989290051137521 11177419

[B9] Banich M. T. (2009). Executive function: the search for an integrated account. Curr. Direct. Psychol. Sci. 18, 89–94

[B10] Banich M. T. Mackiewicz K. L. Depue B. E. Whitmer A. J. Miller G. A. Heller W. (2009). Cognitive control mechanisms, emotion and memory: a neural perspective with implications for psychopathology. Neurosci. Biobehav. Rev. 33, 613–630 10.1016/j.neubiorev.2008.09.010 18948135 PMC2865433

[B11] Bar-Haim Y. (2010). Research review: attention bias modification (ABM). A novel treatment for anxiety disorders. J. Child Psychol. Psychiatry 51, 859–870 10.1111/j.1469-7610.2010.02251.x 20456540

[B12] Bar-Haim Y. Fox N. A. Benson B. Guyer A. E. Williams A. Nelson E. E. (2009). Neural correlates of reward processing in adolescents with a history of inhibited temperament. Psychol. Sci. 20, 1009–1018 10.1111/j.1467-9280.2009.02401.x 19594857 PMC2785902

[B13] Bar-Haim Y. Lamy D. Pergamin L. Bakermans-Kranenburg M. J. van IJzendoorn M. H. (2007). Threat-related attentional bias in anxious and nonanxious individuals: a meta-analytic study. Psychol. Bull. 133, 1–24 10.1037/0033-2909.133.1.1 17201568

[B14] Barlow D. H. (2002). Anxiety and Its Disorders: The Nature and Treatment of Anxiety and Panic, 2nd edn New York, NY: Guilford Press 10.1002/smi.941

[B15] Baxter L. R. Jr. Schwartz J. M. Bergman K. S. Szuba M. P. Guze B. H. Mazziotta J. C. (1992). Caudate glucose metabolic rate changes with both drug and behavior therapy for obsessive-compulsive disorder. Arch. Gen. Psychiatry 49, 681 10.1001/archpsyc.1992.0182009000900 1514872

[B16] Beard C. (2011). Cognitive bias modification for anxiety: current evidence and future directions. Expert Rev. Neurother. 11, 299–311 10.1586/ern.10.194 21306216 PMC3092585

[B17] Beard C. Sawyer A. T. Hofmann S. G. (2012). Efficacy of attention bias modification using threat and appetitive stimuli: a meta-analytic review. Behav. Ther. 43, 724–740 10.1016/j.beth.2012.01.002 23046776 PMC3494088

[B18] Beats B. C. Sahakian B. J. Levy R. (1996). Cognitive performance in tests sensitive to frontal lobe dysfunction in the elderly depressed. Psychol. Med. 26, 591–603 10.1017/S0033291700035662 8733217

[B19] Beck A. T. (1976). Cognitive Therapy and the Emotional Disorders. New York, NY: Meridian 10.1023/A:1005539428336

[B20] Beckmann M. Johansen-Berg H. Rushworth M. F. (2009). Connectivity-based parcellation of human cingulate cortex and its relation to functional specialization. J. Neurosci. 29, 1175–1190 10.1523/JNEUROSCI.3328-08.2009 19176826 PMC6665147

[B21] Bench C. J. Frith C. D. Grasby P. M. Friston K. J. Paulesu E. Frackowiak R. S. J. (1993). Investigations of the functional anatomy of attention using the Stroop test. Neuropsychologia 31, 907–922 10.1016/0028-3932(93)90147-R 8232848

[B22] Berenbaum H. Oltmanns T. F. (1992). Emotional experience and expression in schizophrenia and depression. J. Abnorm. Psychol. 101, 37–44 10.1037/0021-843X.101.1.37 1537971 PMC4370316

[B23] Berenbaum H. Raghavan C. Le H.-N. Vernon L. L. Gomez J. J. (2003). A taxonomy of emotional disturbances. Clin. Psychol. Sci. Pract. 10, 206–226 10.1093/clipsy.bpg011

[B24] Bishop S. J. (2007). Neurocognitive mechanisms of anxiety: an integrative account. Trends Cogn. Sci. 11, 307–316 10.1016/j.tics.2007.05.008 17553730

[B25] Bishop S. J. (2008). Neural mechanisms underlying selective attention to threat. Annal. N.Y. Acad. Sci. 1129, 141–152 10.1196/annals.1417.016 18591476

[B26] Bishop S. J. (2009). Trait anxiety and impoverished prefrontal control of attention. Nat. Neurosci. 12, 92–98 10.1038/nn.2242 19079249

[B27] Bishop S. R. Lau M. Shapiro S. Carlson L. Anderson N. D. Carmody J. (2004). Mindfulness: a proposed operational definition. Clin. Psychol. Sci. Pract. 11, 230–241

[B28] Bradley B. P. Mogg K. Lee S. C. (1997). Attentional biases for negative information in induced and naturally occurring dysphoria. Behav. Res. Ther. 35, 911–927 10.1016/S0005-7967(97)00053-3 9401132

[B28a] Bredemeier K. Berenbaum H. (in press.) Cross-sectional and longitudinal relations between working memory performance and worry. J. Exp. Psychopathol.

[B29] Brehmer Y. Westerberg H. Bäckman L. (2012). Working-memory training in younger and older adults: training gains, transfer, and maintenance. Front. Hum. Neurosci. 6:63 10.3389/fnhum.2012.00063 22470330 PMC3313479

[B30] Breier A. Charney D. S. Heninger G. R. (1985). The diagnostic validity of anxiety disorders and their relationship to depressive illness. Am. J. Psychiatry 142, 787–797 10.1176/appi.pn.2013.5a15 2861752

[B31] Brittlebank A. D. Scott J. Williams J. M. Ferrier I. N. (1993). Autobiographical memory in depression: state or trait marker? Br. J. Psychiatry 162, 118–121 10.1192/bjp.162.1.118 8425125

[B32] Brewer J. A. Worhunsky P. D. Gray J. R. Tang Y. Y. Weber J. Kober H. (2011). Meditation experience is associated with differences in default mode network activity and connectivity. Proc. Natl. Acad. Sci. U.S.A. 108, 20254–20259 10.1073/pnas.1112029108 22114193 PMC3250176

[B33] Brown T. A. Campbell L. A. Lehman C. L. Grisham J. R. Mancill R. B. (2001). Current and lifetime comorbidity of the DSM-IV anxiety and mood disorders in a large clinical sample. J. Abnorm. Psychol. 63, 408–418 10.1037/0021-843X.110.4.585 11727948

[B34] Browning M. Holmes E. A. Murphy S. E. Goodwin G. M. Harmer C. J. (2010). Lateral prefrontal cortex mediates the cognitive modification of attentional bias. Biol. Psychiatry 67, 919–925 10.1016/j.biopsych.2009.10.031 20034617 PMC2866253

[B35] Bruder G. E. Fong R. Tenke C. E. Leite P. Towey J. P. Stewart P. J. (1997). Regional brain asymmetries in major depression with or without an anxiety disorder: a quantitative electroencephalographic study. Biol. Psychiatry 41, 939–948 10.1016/S0006-3223(96)00260-0 9110099

[B36] Bryant R. A. Felmingham K. Whitford T. J. Kemp A. Hughes G. Peduto A. (2008). Rostral anterior cingulate volume predicts treatment response to cognitive-behavioural therapy for posttraumatic stress disorder. J. Psychiatry Neurosci. 33, 142–146 18330460 PMC2265310

[B37] Buck R. (2000). Conceptualizing motivation and emotion. Behav. Brain Sci. 23, 195–196 10.1017/S0140525X00262420

[B38] Butler G. Fennell M. Robson P. Gelder M. (1991). Comparison of behavior therapy and cognitive behavior therapy in the treatment of generalized anxiety disorder. J. Consult. Clin. Psychol. 59, 167–175 10.1037/0022-006X.59.1.167 2002134

[B39] Carrig M. M. Kolden G. G. Strauman T. J. (2009). Using functional magnetic resonance imaging in psychotherapy research: a brief introduction to concepts, methods, and task selection. Psychother. Res. 19, 409–417 10.1080/10503300902735864 19544187

[B40] Carver C. S. (2006). Approach, avoidance, and the self-regulation of affect and action. Motiv. Emot. 30, 105–110 10.1080/00224540903366743 21058573

[B41] Casacalenda N. Perry J. C. Looper K. (2002). Remission in major depressive disorder: a comparison of pharmacotherapy, psychotherapy, and control conditions. Am. J. Psychiatry 159, 1354–1360 10.1176/appi.ajp.159.8.1354 12153828

[B42] Castaneda A. E. Suvisaari J. Marttunen M. Perälä J. Saarni S. I. Aalto-Setälä T. (2010). Cognitive functioning in a population-based sample of young adults with anxiety disorders. Eur. Psychiatry 26, 346–353 10.1016/j.eurpsy.2009.11.006 20627469

[B43] Chambers R. Lo B. C. Y. Allen N. B. (2008). The impact of intensive mindfulness training on attentional control, cognitive style, and affect. Cogn. Ther. Res. 32, 303–322 10.1007/s10608-007-9119-0

[B44] Chein J. M. Morrison A. B. (2010). Expanding the mind's workspace: training and transfer effects with a complex working memory span task. Psychon. Bull. Rev. 17, 193–199 10.3758/PBR.17.2.193 20382919

[B45] Chiew K. S. Braver T. S. (2011). Positive affect versus reward: emotional and motivational influences on cognitive control. Front. Psychol. 2:279 10.3389/fpsyg.2011.00279 22022318 PMC3196882

[B46] Cisler J. M. Bacon A. K. Williams N. L. (2009). Phenomenological characteristics of attentional biases toward threat: a critical review. Cogn. Ther. Res. 33, 221–234 10.1007/s10608-007-9161-y 20622985 PMC2901130

[B47] Clark D. A. Beck A. T. (2010). Cognitive theory and therapy of anxiety and depression: convergence with neurobiological findings. Trends Cogn. Sci. 14, 418–424 10.1016/j.tics.2010.06.007 20655801

[B48] Clark L. A. (1989). The anxiety and depressive disorders: descriptive psychopathology and differential diagnosis, in Anxiety and Depression: Distinctive and Overlapping Features, eds Kendall P. C. Watson D. (San Diego, CA: Academic Press), 83–129

[B49] Collette F. Hogge M. Salmon E. Van der Linden M. (2006). Exploration of the neural substrates of executive functioning by functional neuroimaging. Neuroscience 139, 209–221 10.1016/j.neuroscience.2005.05.035 16324796

[B50] Collette F. Van der Linden M. Laureys S. Delfiore G. Degueldre C. Luxen A. (2005). Exploring the unity and diversity of the neural substrates of executive functioning. Hum. Brain Mapp. 25, 409–423 10.1002/hbm.20118 15852470 PMC6871684

[B51] Compton R. J. Banich M. T. Mohanty A. Milham M. P. Herrington J. Miller G. A. (2003). Paying attention to emotion: an fMRI investigation of cognitive and emotional Stroop tasks. Cogn. Affect. Behav. Neurosci. 3, 81–96 10.3758/CABN.3.2.81 12943324

[B52] Corbetta M. Patel G. Shulman G. L. (2008). The reorienting system of the human brain: from environment to theory of mind. Neuron 58, 306–324 10.1016/j.neuron.2008.04.017 18466742 PMC2441869

[B53] Costafreda S. G. Chu C. Ashburner J. Fu C. H. Y. (2009). Prognostic and diagnostic potential of the structural neuroanatomy of depression. PLoS ONE 4:e6353 10.1371/journal.pone.0006353 19633718 PMC2712086

[B54] Dahlin E. Neely A. S. Larsson A. Bäckman L. Nyberg L. (2008). Transfer of learning after updating training mediated by the striatum. Science 320, 1510–1512 10.1126/science.1155466 18556560

[B55] Dalgleish T. Spinks H. Yiend J. Kuyken W. (2001). Autobiographical memory style in seasonal affective disorder and its relationship to future symptom remission. J. Abnorm. Psychol. 110, 335–340 10.1037/0021-843X.110.2.335 11358027

[B56] Damsa C. Kosel M. Moussally J. (2009). Current status of brain imaging in anxiety disorders. Curr. Opin. Psychiatry 22, 96–110 10.1097/YCO.0b013e328319bd10 19122541

[B57] Dandeneau S. D. Baldwin M. W. Baccus J. R. Sakellaropoulo M. Pruessner J. C. (2007). Cutting stress off at the pass: reducing vigilance and responsiveness to social threat by manipulating attention. J. Pers. Soc. Psychol. 93, 651–666 10.1037/0022-3514.93.4.651 17892337

[B58] Danti S. Ricciardi E. Gentili C. Gobbini M. I. Pietrini P. Guazzelli M. (2010). Is social phobia a “mis-communication” disorder? Brain functional connectivity during face perception differs between patients with social phobia and healthy control subjects. Front. Syst. Neurosci. 4:152 10.3389/fnsys.2010.00152 21152341 PMC2996271

[B59] Davidson R. J. Henriques J. B. (2000). Regional brain function in sadness and depression, in The Neuropsychology of Emotion, ed Borod J. (New York, NY: Oxford University Press), 269–297 10.1016/S0959-4388(99)80032-4

[B60] Davidson R. J. Irwin W. Anderle M. J. Kalin N. H. (2003). The neural substrates of affective processing in depressed patients treated with venlafaxine. Am. J. Psychiatry 160, 64–75 10.1176/appi.ajp.160.1.64 12505803

[B61] Delaveau P. Jabourian M. Lemogne C. Guionnet S. Bergouignan L. Fossati P. (2011). Brain effects of antidepressants in major depression: a meta-analysis of emotional processing studies. J. Affect. Disord. 130, 66–74 10.1016/j.jad.2010.09.032 21030092

[B62] Derakshan N. Eysenck M. W. (1998). Working memory capacity in high trait-anxious and repressor groups. Cogn. Emot. 12, 697–713 10.1080/026999398379501

[B63] Derakshan N. Eysenck M. W. (2009). Anxiety, processing efficiency, and cognitive performance: new developments from attentional control theory. Eur. Psychol. 14, 168–176 10.1037/1528-3542.7.2.336

[B64] DeRubeis R. J. Gelfand L. A. Tang T. Z. Simons A. D. (1999). Medications versus cognitive behavior therapy for severely depressed outpatients: mega-analysis of four randomized comparisons. Am. J. Psychiatry 156, 1007–1013 10401443 10.1176/ajp.156.7.1007

[B65] DeRubeis R. J. Siegle G. J. Hollon S. D. (2008). Cognitive therapy versus medication for depression: treatment outcomes and neural mechanisms. Nat. Rev. Neurosci. 9 788–796 10.1038/nrn2345 18784657 PMC2748674

[B66] Desbordes G. Negi L. T. Pace T. W. Wallace B. A. Raison C. L. Schwartz E. L. (2012). Effects of mindful-attention and compassion meditation training on amygdala response to emotional stimuli in an ordinary, non-meditative state. Front. Hum. Neurosci. 6:292 10.3389/fnhum.2012.00292 23125828 PMC3485650

[B67] D'Esposito M. (2001). Functional neuroimaging of working memory, in Handbook of Functional Neuroimaging of Cognition, eds Cabeza R. Kingstone A. (Cambridge, MA: The MIT Press), 293–327 10.1037/0894-4105.20.5.497

[B68] Dolcos F. Iordan A. D. Dolcos S. (2011). Neural correlates of emotion–cognition interactions: a review of evidence from brain imaging investigations. J. Cogn. Psychol. 23, 669–694 10.1080/20445911.2011.594433 22059115 PMC3206704

[B69] Dunkin J. J. Leuchter A. F. Cook I. A. Kasl-Godley J. E. Abrams M. Rosenberg-Thompson S. (2000). Executive dysfunction predicts nonresponse to fluoxetine in major depression. J. Affect. Disord. 60, 13–23 10.1016/S0165-0327(99)00157-3 10940443

[B70] Elbert T. Pantev C. Wienbruch C. Rockstroh B. Taub E. (1995). Increased cortical representation of the fingers of the left hand in string players. Science 270, 305–307 10.1126/science.270.5234.305 7569982

[B71] Elliott R. Sahakian B. J. Herrod J. J. Robbins T. W. Paykel E. S. (1997). Abnormal response to negative feedback in unipolar depression: evidence for a diagnosis specific impairment. J. Neurol. Neurosurg. Psychiatry 63, 74–82 9221971 10.1136/jnnp.63.1.74 PMC2169625

[B72] Elliott R. Sahakian B. J. McKay A. P. Herrod J. J. Robbins T. W. Paykel E. S. (1996). Neuropsychological impairments in unipolar depression: the influence of perceived failure on subsequent performance. Psychol. Med. 26, 975–989 10.1017/S0033291700035303 8878330

[B73] Engelmann J. B. Damaraju E. Padmala S. Pessoa L. (2009). Combined effects of attention and motivation on visual task performance: transient and sustained motivational effects. Front. Hum. Neurosci. 3:4 10.3389/neuro.09.004.2009 19434242 PMC2679199

[B74] Engelmann J. B. Pessoa L. (2007). Motivation sharpens exogenous spatial attention. Emotion 7, 668–674 10.1037/1528-3542.7.3.668 17683222

[B75] Engels A. S. Heller W. Mohanty A. Herrington J. D. Banich M. T. Webb A. G. (2007). Specificity of regional brain activity in anxiety types during emotion processing. Psychophysiology 44, 352–363 10.1111/j.1469-8986.2007.00518.x 17433094

[B76] Engels A. S. Heller W. Spielberg J. M. Warren S. L. Sutton B. P. Banich M. T. (2010). Co-occurring anxiety influences patterns of brain activity in depression. Cogn. Affect. Behav. Neurosci. 10, 141–156 10.3758/CABN.10.1.141 20233962 PMC4403735

[B77] Erickson K. I. Colcombe S. J. Wadhwa R. Bherer L. Peterson M. S. Scalf P. E. (2007). Training-induced functional activation changes in dual-task processing: an fMRI study. Cereb. Cortex 17, 192–204 10.1093/cercor/bhj137 16467562

[B78] Eysenck M. W. (1985). Anxiety and cognitive-task performance. Pers. Individ. Differ. 6, 579–586 10.1016/0191-8869(85)90007-8

[B79] Eysenck M. W. Derakshan N. (2011). New perspectives in attentional control theory. Pers. Individ. Differ. 50, 955–960 10.1111/j.1467-8624.2012.01738.x 22364371

[B80] Eysenck M. W. Derakshan N. Santos R. Calvo M. G. (2007). Anxiety and cognitive performance: attentional control theory. Emotion 7, 336–353 10.1037/1528-3542.7.2.336 17516812

[B81] Eysenck M. W. Payne S. Derakshan N. (2005). Trait anxiety, visuospatial processing, and working memory. Cogn. Emot. 19, 1214–1228 10.1080/0269993050026024518686056

[B82] Felmingham K. Kemp A. Williams L. Das P. Hughes G. Trait anxiety (2007). Changes in anterior cingulate and amygdala after cognitive behavior therapy of posttraumatic stress disorder. Psychol. Sci. 18, 127–129 10.1111/j.1467-9280.2007.01860.x 17425531

[B83] Fernandes L. O. L. Miller G. A. (1995). Compromised performance and abnormal psychophysiology associated with the Wisconsin Psychosis-Proneness Scales, in The Behavioral High-Risk Paradigm in Psychopathology, ed Miller G. A. (New York, NY: Springer-Verlag), 47–87 10.1007/978-1-4612-4234-5_2

[B83a] Fitzgerald P. B. Laird A. R. Maller J. Daskalakis Z. J. (2008). A meta-analytic study of changes in brain activation in depression. Hum. Brain Mapp. 29, 683–695 10.1002/hbm.20426 17598168 PMC2873772

[B84] Frewen P. A. Dozois D. J. A. Lanius R. A. (2008). Neuroimaging studies of psychological interventions for mood and anxiety disorders: empirical and methodological review. Clin. Psychol. Rev. 28, 228–246 10.1016/j.cpr.2007.05.002 17602811

[B85] Fu C. H. Steiner H. Costafreda S. G. (2012). Predictive neural biomarkers of clinical response in depression: a meta-analysis of functional and structural neuroimaging studies of pharmacological and psychological therapies. Neurobiol. Dis. 52, 75–83 10.1016/j.nbd.2012.05.008 22659303

[B86] Fu C. H. Williams S. C. Cleare A. J. Brammer M. J. Walsh N. D. Kim J. (2004). Attenuation of the neural response to sad faces in major depression by antidepressant treatment: a prospective, event-related functional magnetic resonance imaging study. Arch. Gen. Psychiatry 61, 877 10.1001/archpsyc.61.9.877 15351766

[B87] Furmark T. Appel L. Michelgård Å. Wahlstedt K. Åhs F. Zancan S. (2005). Cerebral blood flow changes after treatment of social phobia with the neurokinin-1 antagonist GR205171, citalopram, or placebo. Biol. Psychiatry 58, 132–142 10.1016/j.biopsych.2005.03.029 16038684

[B88] Furmark T. Tillfors M. Marteinsdottir I. Fischer H. Pissiota A. Långstrom B. (2002). Common changes in cerebral blood flow in patients with social phobia treated with citalopram or cognitive-behavioral therapy. Arch. Gen. Psychiatry 59, 425 10.1001/archpsyc.59.5.425 11982446

[B89] Gersh F. S. Fowles D. C. (1979). Neurotic depression: the concept of anxious depression, in The Psychobiology of the Depressive Disorders: Implications for the Effects of Stress, ed Depue R. A. (New York, NY: Academic Press), 81–104

[B90] Goeleven E. De Raedt R. Baert S. Koster E. H. W. (2006). Deficient inhibition of emotional information in depression. J. Affect. Disord. 93, 149–157 10.1016/j.jad.2006.03.007 16647141

[B91] Goldapple K. Segal Z. Garson C. Lau M. Bieling P. Kennedy S. (2004). Modulation of cortical-limbic pathways in major depression: treatment-specific effects of cognitive behavior therapy. Arch. Gen. Psychiatry 61, 34–41 10.1001/archpsyc.61.1.34 14706942

[B92] Goldin P. R. Gross J. J. (2010). Effects of mindfulness-based stress reduction (MBSR) on emotion regulation in social anxiety disorder. Emotion 10, 83–91 10.1037/a0018441 20141305 PMC4203918

[B93] Goldin P. R. Manber T. Hakimi S. Canli T. Gross J. J. (2009). Neural bases of social anxiety disorder: emotional reactivity and cognitive regulation during social and physical threat. Arch. Gen. Psychiatry 66, 170–180 10.1001/archgenpsychiatry.2008.525 19188539 PMC4142809

[B94] Gotlib I. H. Joormann J. (2010). Cognition and depression: current status and future directions. Ann. Rev. Clin. Psychol. 6, 285–312 10.1146/annurev.clinpsy.121208.131305 20192795 PMC2845726

[B95] Gotlib I. H. Krasnoperova E. Yue D. N. Joormann J. (2004). Attentional biases for negative interpersonal stimuli in clinical depression. J. Abnorm. Psychol. 113, 127–135 10.1037/0021-843X.113.1.121 14992665

[B96] Gould R. A. Otto M. W. Pollack M. H. (1995). A meta-analysis of treatment outcome for panic disorder. Clin. Psychol. Rev. 15, 819–844 10.1016/0272-7358(95)00048-8

[B97] Gould R. A. Otto M. W. Pollack M. H. Yap L. (1997). Cognitive behavioral and pharmacological treatment of generalized anxiety disorder: a preliminary meta-analysis. Behav. Ther. 28, 285–305 10.1016/S0005-7894(97)80048-2

[B98] Gray J. A. (1975). Elements of a Two-Process Theory of Learning. Oxford, England: Academic Press

[B99] Gray J. A. (1982). The Neuropsychology of Anxiety: An Enquiry into the Functions of the Septo-Hippocampal System. New York, NY: Oxford University Press

[B100] Gray J. R. (2004). Integration of emotion and cognitive control. Curr. Dir. Psychol. Sci. 13, 46–48 10.1111/j.0963-7214.2004.00272.x

[B101] Gray J. R. Braver T. S. Raichle M. E. (2002). Integration of emotion and cognition in the lateral prefrontal cortex. Proc. Natl. Acad. Sci. U.S.A. 99, 4115–4120 10.1073/pnas.062381899 11904454 PMC122657

[B102] Guyer A. E. Choate V. R. Detloff A. Benson B. Nelson E. E. Perez-Edgar K. (2012). Striatal functional alteration during incentive anticipation in pediatric anxiety disorders. Am. J. Psychiatry 169, 205 10.1176/appi.ajp.2011.11010006 22423352 PMC3307369

[B103] Guyer A. E. Nelson E. E. Perez-Edgar K. Hardin M. G. Roberson-Nay R. Monk C. S. (2006). Striatal functional alteration in adolescents characterized by early childhood behavioral inhibition. J. Neurosci. 26, 6399–6405 10.1523/JNEUROSCI.0666-06.2006 16775126 PMC6674047

[B104] Hahn A. Stein P. Windischberger C. Weissenbacher A. Spindelegger C. Moser E. (2011). Reduced resting-state functional connectivity between amygdala and orbitofrontal cortex in social anxiety disorder. Neuroimage 56, 881–889 10.1016/j.neuroimage.2011.02.064 21356318

[B105] Hakamata Y. Lissek S. Bar-Haim Y. Britton J. C. Fox N. A. Leibenluft E. (2010). Attention bias modification treatment: a meta-analysis toward the establishment of novel treatment for anxiety. Biol. Psychiatry 68, 982–990 10.1016/j.biopsych.2010.07.021 20887977 PMC3296778

[B106] Hallion L. S. Ruscio A. M. (2011). A meta-analysis of the effect of cognitive bias modification on anxiety and depression. Psychol. Bull. 137, 940–958 10.1037/a0024355 21728399

[B107] Hardin M. G. Perez-Edgar K. Guyer A. E. Pine D. P. Fox N. A. Ernst M. (2006). Reward and punishment sensitivity in shy and non-shy adults: relations between social and motivated behavior. Pers. Individ. Differ. 40, 699–711 10.1016/j.paid.2005.08.010 19718273 PMC2733521

[B108] Hardin M. G. Schroth E. Pine D. S. Ernst M. (2007). Incentive-related modulation of cognitive control in healthy, anxious, and depressed adolescents: development and psychopathology related differences. J. Child Psychol. Psychiatry 48, 446–454 10.1111/j.1469-7610.2006.01722.x 17501725 PMC2737725

[B109] Heller A. S. Johnstone T. Shackman A. J. Light S. N. Peterson M. J. Kolden G. G. (2009). Reduced capacity to sustain positive emotion in major depression reflects diminished maintenance of frontostriatal brain activation. Proc. Natl. Acad. Sci. U.S.A. 106, 22445–22450 10.1073/pnas.0910651106 20080793 PMC2796908

[B110] Heller W. (1993). Neuropsychological mechanisms of individual differences in emotion, personality, and arousal. Neuropsychology 7, 476–489

[B111] Heller W. Koven N. S. Miller G. A. (2003). Regional brain activity in anxiety and depression, cognition/emotion interaction, and emotion regulation, in The Asymmetrical Brain, eds Hugdahl K. Davidson R. J. (Cambridge, MA: MIT), 533–564

[B112] Heller W. Nitschke J. B. (1997). Regional brain activity in emotion: a framework for understanding cognition in depression. Cogn. Emot. 11, 637–661 10.1080/026999397379845a

[B113] Henriques J. B. Davidson R. J. (2000). Decreased responsiveness to reward in depression. Cogn. Emot. 14, 711–724 10.1080/02699930050117684

[B114] Hertel P. T. Brozovich F. (2010). Cognitive habits and memory distortions in anxiety and depression. Curr. Dir. Psychol. Sci. 19, 155–160 10.1177/0963721410370137

[B115] Herrington J. D. Heller W. Mohanty A. Engels A. S. Banich M. T. Webb A. G. (2010). Localization of asymmetric brain function in emotion and depression. Psychophysiology 47, 442–454 10.1111/j.1469-8986.2009.00958.x 20070577 PMC3086589

[B116] Herrington J. D. Mohanty A. Koven N. S. Fisher J. E. Stewart J. L. Banich M. T. (2005). Emotion-modulated performance and activity in left dorsolateral prefrontal cortex. Emotion 5, 200–207 10.1037/1528-3542.5.2.200 15982085

[B117] Hertel P. T. Brozovich F. (2010). Cognitive habits and memory distortions in anxiety and depression. Curr. Dir. Psychol. Sci. 19, 155–160 10.1177/0963721410370137

[B118] Hertel P. T. Mathews A. (2011). Cognitive bias modification: past perspectives, current findings, and future applications. Perspect. Psychol. Sci. 6, 521–536 10.1177/1745691611421205 26168375

[B119] Hirsch C. R. Hayes S. Mathews A. (2009). Looking on the bright side: accessing benign meanings reduces worry. J. Abnorm. Psychol. 118, 44–54 10.1037/a0013473 19222313

[B120] Hofmann S. G. Sawyer A. T. Witt A. A. Oh D. (2010). The effect of mindfulness-based therapy on anxiety and depression: a meta-analytic review. J. Consult. Clin. Psychol. 78, 169–183 10.1037/a0018555 20350028 PMC2848393

[B121] Hollon S. D. Stewart M. O. Strunk D. (2006). Enduring effects for cognitive behavior therapy in the treatment of depression and anxiety. Ann. Rev. Psychol. 57, 285–315 10.1146/annurev.psych.57.102904.190044 16318597

[B122] Holmes A. J. Pizzagalli D. A. (2007). Task feedback effects on conflict monitoring and executive control: relationship to subclinical measures of depression. Emotion 7, 68–76 10.1037/1528-3542.7.1.68 17352564 PMC3196340

[B123] Holmes A. J. Pizzagalli D. A. (2008). Spatiotemporal dynamics of error processing dysfunctions in major depressive disorder. Arch. Gen. Psychiatry 65, 179–188 10.1001/archgenpsychiatry.2007.19 18250256 PMC2587280

[B124] Holzel B. K. Lazar S. W. Gard T. Schuman-Olivier Z. Vago D. R. Ott U. (2011). How does mindfulness meditation work? Proposing mechanisms of action from a conceptual and neural perspective. Perspect. Psychol. Sci. 6, 537–559 10.1177/1745691611419671 26168376

[B125] Ives-Deliperi V. L. Solms M. Meintjes E. M. (2011). The neural substrates of mindfulness: an fMRI investigation. Soc. Neurosci. 6, 231–242 10.1080/17470919.2010.513495 20835972

[B126] Jaeggi S. M. Buschkuehl M. Jonides J. Shah P. (2011). Short- and long-term benefits of cognitive training. Proc. Natl. Acad. Sci. U.S.A. 105, 6829–6833 10.1073/pnas.1103228108 21670271 PMC3121868

[B127] Jazbec S. McClure E. Hardin M. Pine D. S. Ernst M. (2005). Cognitive control under contingencies in anxious and depressed adolescents: an antisaccade task. Biol. Psychiatry 58, 632–639 10.1016/j.biopsych.2005.04.010 16018983

[B128] Jimura K. Locke H. S. Braver T. S. (2010). Prefrontal cortex mediation of cognitive enhancement in rewarding motivational contexts. Proc. Natl. Acad. Sci. U.S.A. 107, 8871–8876 10.1073/pnas.1002007107 20421489 PMC2889311

[B129] Johnson D. R. (2009). Emotional attention set-shifting and its relationship to anxiety and emotion regulation. Emotion 9, 681–690 10.1037/a0017095 19803590

[B130] Jonides J. (2004). How does practice makes perfect? Nat. Neurosci. 7, 10–11 10.1038/nn0104-10 14699412

[B131] Joormann J. (2010). Cognitive inhibition and emotion regulation in depression. Curr. Direct. Psychol. Sci. 19, 161–166 10.1177/0963721410370293

[B132] Joormann J. Gotlib I. H. (2008). Updating the contents of working memory in depression: interference from irrelevant negative material. J. Abnorm. Psychol. 117, 182–192 10.1037/0021-843X.117.1.182 18266496

[B133] Judd L. L. Kessler R. C. Paulus M. P. Zeller P. V. Wittchen H. U. Kunovac J. L. (1998). Comorbidity as a fundamental feature of generalized anxiety disorders: results from the National Comorbidity Study (NCS). Acta Psychiatr. Scand. 98, 6–11 9777041 10.1111/j.1600-0447.1998.tb05960.x

[B134] Kane M. J. Engle R. W. (2002). The role of prefrontal cortex in working-memory capacity, executive attention, and general fluid intelligence: an individual-differences perspective. Psychon. Bull. Rev. 9, 637–671 10.3758/BF03196323 12613671

[B135] Keller J. Nitschke J. B. Bhargava T. Deldin P. J. Gergen J. A. Miller G. A. (2000a). Neuropsychological differentiation of depression and anxiety. J. Abnorm. Psychol. 109, 3–10 10.1037/0021-843X.109.1.3 10740930

[B136] Keller M. B. McCullough J. P. Klein D. N. Arnow B. Dunner D. L. Gelenberg A. J. (2000b). A comparison of nefazodone, the cognitive behavioral-analysis system of psychotherapy, and their combination for the treatment of chronic depression. New Engl. J. Med. 342, 1462–1470 10.1056/NEJM200005183422001 10816183

[B137] Kendall P. C. Sugarman A. (1997). Attrition in the treatment of childhood anxiety disorders. J. Consult. Clin. Psychol. 65, 883 10.1037/0022-006X.65.5.883 9337507

[B138] Kentgen L. M. Tenke C. E. Pine D. S. Fong R. Klein R. G. Bruder G. E. (2000). Electroencephalographic asymmetries in adolescents with major depression: influence of comorbidity with anxiety disorders. J. Abnorm. Psychol. 109, 797–802 10.1037/0021-843X.109.4.797 11196007

[B139] Kessler R. C. McGonagle K. A. Zhao S. Nelson C. B. Hughes M. Eshleman S. (1994). Lifetime and 12-month prevalence of DSM-III-R psychiatric disorders in the United States: results from the National Comorbidity Survey. Arch. Gen. Psychiatry 51, 8–19 10.1001/archpsyc.1994.03950010008002 8279933

[B140] Klingberg T. (2006). Development of a superior frontal? intraparietal network for visuo-spatial working memory. Neuropsychologia 44, 2171–2177 10.1016/j.neuropsychologia.2005.11.019 16405923

[B141] Klingberg T. Fernell E. Olesen P. J. Johnson M. Gustafsson P. Dahlstrom K. (2005). Computerized training of working memory in children with ADHD- a randomized, controlled trial. J. Am. Acad. Child Adolesc. Psychiatry 44, 177–186 10.1097/00004583-200502000-00010 15689731

[B142] Knutson B. Bhanji J. P. Cooney R. E. Atlas L. Y. Gotlib I. H. (2008). Neural responses to monetary incentives in major depression. Biol. Psychiatry 63, 686–692 10.1016/j.biopsych.2007.07.023 17916330 PMC2290738

[B143] Koechlin E. Hyafil A. (2007). Anterior prefrontal function and the limits of human decision-making. Science 318, 594–598 10.1126/science.1142995 17962551

[B144] Kolb B. Whishaw I. Q. (1998). Brain plasticity and behavior. Ann. Rev. Psychol. 49, 43–64 10.1146/annurev.psych.49.1.43 9496621

[B145] Koster E. H. W. Fox E. MacLeod C. (2009). Introduction to the special section on cognitive bias modification in emotional disorders. J. Abnorm. Psychol. 118, 1–4 10.1037/a0014379 19222308

[B146] Kouneiher F. Charron S. Koechlin E. (2009). Motivation and cognitive control in the human prefrontal cortex. Nat. Neurosci. 12, 939–945 10.1038/nn.2321 19503087

[B147] Kozak M. J. Miller G. A. (1982). Hypothetical constructs versus intervening variables: a re-appraisal of the three-systems model of anxiety assessment. Behav. Assess. 4, 347–358

[B148] Kumari V. (2006). Do psychotherapies produce neurobiological effects? Acta Neuropsychiatr. 18, 61–70 10.1111/j.1601-5215.2006.00127.x 26989794

[B149] Kuyken W. Watkins E. Beck A. T. (2005). Cognitive behavior therapy for mood disorders, in Oxford Textbook of Psychotherapy, eds Gabbard G. O. Beck J. S. Holmes J. (New York, NY: Oxford University Press), 111–126 10.1016/j.psc.2010.04.005

[B150] Laming D. R. (2000). On the behavioural interpretation of neurophysiological observation. Behav. Brain Sci. 23, 209

[B151] Lang P. J. (1968). Fear reduction and fear behavior: problems in treating a construct, in Research in Psychotherapy Conference (Chicago, IL: American Psychological Association), 3rd May–June 1966. 10.1037/10546-004

[B152] Lang P. J. Melamed B. G. Hart J. (1970). A psychophysiological analysis of fear modification using an automated desensitization procedure. J. Abnorm. Psychol., 229–234 10.1037/h0029875 5483369

[B153] Lazar S. W. Kerr C. E. Wasserman R. H. Gray J. R. Greve D. N. Treadway M. T. (2005). Meditation experience is associated with increased cortical thickness. Neuroreport 16, 1893–1897 10.1097/01.wnr.0000186598.66243.19 16272874 PMC1361002

[B154] Levin R. L. Heller W. Mohanty A. Herrington J. D. Miller G. A. (2007). Cognitive deficits in depression and functional specificity of regional brain activity. Cogn. Ther. Res. 31, 211–233 10.1007/s10608-007-9128-z

[B155] Liggan D. Y. Kay J. (1999). Some neurobiological aspects of psychotherapy: a review. J. Psychother. Pract. Res. 8, 103 10079458 PMC3330538

[B156] Linden D. E. J. (2006). How psychotherapy changes the brain- the contribution of functional neuroimaging. Mol. Psychiatry 11, 528–538 10.1038/sj.mp.4001816 16520823

[B157] Locke H. S. Braver T. S. (2008). Motivational influences on cognitive control: behavior, brain activation, and individual differences. Cogn. Affect. Behav. Neurosci. 8, 99–112 10.3758/CABN.8.1.99 18405050

[B158] Luders E. Clark K. Narr K. L. Toga A. W. (2011). Enhanced brain connectivity in long-term meditation practitioners. Neuroimage 57, 1308–1316 10.1016/j.neuroimage.2011.05.075 21664467 PMC3176828

[B159] Luders E. Kurth F. Mayer E. A. Toga A. W. Narr K. L. Gaser C. (2012). The unique brain anatomy of meditation practitioners: alterations in cortical gyrification. Front. Hum. Neurosci. 6:34 10.3389/fnhum.2012.00034 22393318 PMC3289949

[B160] Luders E. Toga A. W. Lepore N. Gaser C. (2009). The underlying anatomical correlates of long-term meditation: larger hippocampal and frontal volumes of gray matter. Neuroimage 45, 672–678 10.1016/j.neuroimage.2008.12.061 19280691 PMC3184843

[B161] Lydiard R. B. Brawman-Mintzer O. (1998). Anxious depression. J. Clin. Psychiatry 59, 10–17 9840193

[B162] MacDonald A. W. Jones J. A. H. (2009). Functional imaging in clinical assessment? The rise of neurodiagnostics with fMRI, in Oxford Handbook of Personality Assessment ed Butcher J. N. (New York, NY: Oxford University Press), 364–374 10.1093/oxfordhb/9780195366877.013.0019

[B163] MacLeod C. (2012). Cognitive bias modification procedures in the management of mental disorders. Curr. Opin. Psychiatry 25, 114 10.1097/YCO.0b013e32834fda4a 22227631

[B164] MacLeod C. Donnelan A. M. (1993). Individual differences in anxiety and the restriction of working memory capacity. Pers. Individ. Differ. 15, 163–173 10.1016/0191-8869(93)90023-V

[B165] MacLeod C. Rutherford E. M. Campbell L. Ebsworthy G. Holker L. (2002). Selective attention and emotional vulnerability: assessing the causal basis of their association through the experimental induction of attentional bias. J. Abnorm. Psychol. 111, 107–123 10.1037/0021-843X.111.1.107 11866165

[B166] Malykhin N. V. Carter R. Seres P. Coupland N. J. (2010). Structural changes in the hippocampus in major depressive disorder: contributions of disease and treatment. J. Psychiatry Neurosci. 35, 337 10.1503/jpn.100002 20731966 PMC2928287

[B167] Maner J. K. Schmidt N. B. (2006). The role of risk avoidance in anxiety. Behav. Ther. 37, 181–189 10.1016/j.beth.2005.11.003 16942970

[B168] Manoach D. S. Halpern E. F. Kramer T. S. Chang Y. Goff D. C. Rauch S. L. (2001). Test-retest reliability of a functional MRI working memory paradigm in normal and schizophrenic subjects. Am. J. Psychiatry 158, 955–958 10.1176/appi.ajp.158.6.955 11384907

[B169] Manji H. K. Quiroz J. A. Sporn J. Payne J. L. Denicoff K. A. Gray N. (2003). Enhancing neuronal plasticity and cellular resilience to develop novel, improved therapeutics for difficult-to-treat depression. Biol. Psychiatry 53, 707–742 10.1016/S0006-3223(03)00117-3 12706957

[B170] Martell C. R. Addis M. E. Jacobson N. S. (2001). Depression in Context: Strategies for Guided Action. New York, NY: Norton

[B171] Mathews A. MacLeod C. (2005). Cognitive vulnerability to emotional disorders. Annu. Rev. Clin. Psychol. 1, 167–195 10.1146/annurev.clinpsy.1.102803.143916 17716086

[B172] Mayberg H. S. (1997). Limbic-cortical dysregulation: a proposed model of depression. J. Neuropsychiatry Clin. Neurosci. 9, 471–481 10.1176/appi.pn.2013.5a10 9276848

[B173] Mayberg H. S. (2000). Brain mapping: depression, in Brain Mapping: The Diseases A. W. Toga, J. C. Mazziotta, and R. C. Frackowiak (San Diego, CA: Academic Press), 485–507

[B174] Mayberg H. S. (2003). Modulating dysfunctional limbic-cortical circuits in depression: towards development of brain-based algorithms for diagnosis and optimized treatment. Br. Med. Bull. 65, 193–207 10.1093/bmb/65.1.193 12697626

[B175] Mayberg H. S. Brannan S. K. Mahurin R. K. Jerabek P. A. Brickman J. S. Tekell J. L. (1997). Cingulate function in depression: a potential predictor of treatment response. Neuroreport 8, 1057–1061 9141092 10.1097/00001756-199703030-00048

[B176] Mayberg H. S. Brannan S. K. Tekell J. L. Silva J. A. Mahurin R. K. McGinnis S. (2000). Regional metabolic effects of fluoxetine in major depression: serial changes and relationship to clinical response. Biol. Psychiatry 48, 830–843 10.1016/S0006-3223(00)01036-2 11063978

[B177] Mayberg H. S. Liotti M. Brannan S. K. McGinnis S. Mahurin R. K. Jerabek P. A. (1999). Reciprocal limbic–cortical function and negative mood: converging PET findings in depression and normal sadness. Am. J. Psychiatry 156, 675–682 10327898 10.1176/ajp.156.5.675

[B178] Mayberg H. S. Lozano A. M. Voon V. McNeely H. E. Seminowicz D. Hamani C. (2005). Deep brain stimulation for treatment-resistant depression. Neuron 45, 651–660 10.1016/j.neuron.2005.02.014 15748841

[B179] McCabe S. B. Gotlib I. H. (1995). Selective attention and clinical depression: performance on a deployment-of-attention task. J. Abnorm. Psychol. 104, 241–245 10.1037/0021-843X.104.1.241 7897048

[B180] McClure E. B. Adler A. Monk C. S. Cameron J. Smith S. Nelson E. E. (2007). fMRI predictors of treatment outcome in pediatric anxiety disorders. Psychopharmacology 191, 97–105 10.1007/s00213-006-0542-9 16972100

[B181] McNally R. J. (1998). Information-processing abnormalities in anxiety disorders: implications for cognitive neuroscience. Cogn. Emot. 12, 479–495 10.1080/026999398379682

[B182] Miller G. A. (1996). Presidential address: how we think about cognition, emotion, and biology in psychopathology. Psychophysiology 33, 615–628 10.1111/j.1469-8986.1996.tb02356.x 8961782

[B183] Miller G. A. (2010). Mistreating psychology in the decades of the brain. Perspect. Psychol. Sci. 5, 716–743 10.1177/1745691610388774 21949539 PMC3177535

[B184] Miller G. A. Crocker L. D. Spielberg J. M. Infantolino Z. P. Heller W. (2013). Issues in localization of brain function: the case of lateralized frontal cortex in cognition, emotion, and psychopathology. Front. Integr. Neurosci. 7:2 10.3389/fnint.2013.00002 23386814 PMC3558680

[B185] Miller G. A. Elbert T. Sutton B. P. Heller W. (2007). Innovative clinical assessment technologies: challenges and opportunities in neuroimaging. Psychol. Assess. 19, 58–73 10.1037/1040-3590.19.1.58 17371123

[B186] Miyake A. Friedman N. P. (2012). The nature and organization of individual differences in executive functions: four general conclusions. Curr. Direct. Psychol. Sci. 21, 8–14 10.1177/0963721411429458 22773897 PMC3388901

[B187] Miyake A. Friedman N. P. Emerson M. J. Witzki A. H. Howerter A. Wager T. D. (2000). The unity and diversity of executive functions and their contributions to complex “frontal lobe” tasks: a latent variable analysis. Cogn. Psychol. 41, 49–100 10.1006/cogp.1999.0734 10945922

[B188] Mohanty A. Engels A. S. Herrington J. D. Heller W. Ho R. M. Banich M. T. (2007). Differential engagement of anterior cingulate cortex subdivisions for cognitive and emotional function. Psychophysiology 44, 343–351 10.1111/j.1469-8986.2007.00515.x 17433093

[B189] Mohlman J. (2008). More power to the executive? A preliminary test of CBT plus executive skills training for treatment of late-life GAD. Cogn. Behav. Pract. 15, 306–316 10.1016/j.cbpra.2007.07.002

[B190] Mohlman J. Gorman J. M. (2005). The role of executive functioning in CBT: a pilot study with anxious older adults. Behav. Res. Ther. 43, 447–465 10.1016/j.brat.2004.03.007 15701356

[B191] Monroe S. M. Reid M. W. (2009). Life stress and major depression. Curr. Direct. Psychol. Sci. 18, 68–72 10.1111/j.1467-8721.2009.01611.x

[B192] Moore A. Gruber T. Derose J. Malinowski P. (2012). Regular, brief mindfulness meditation practice improves electrophysiological markers of attentional control. Front. Hum. Neurosci. 6:18 10.3389/fnhum.2012.00018 22363278 PMC3277272

[B193] Murphy F. C. Michael A. Robbins T. W. Sahakian B. J. (2003). Neuropsychological impairment in patients with major depressive disorder: the effects of feedback on task performance. Psychol. Med. 33, 455–467 12701666 10.1017/s0033291702007018

[B194] Nakatani E. Nakgawa A. Ohara Y. Goto S. Uozumi N. Iwakin M. (2003). Effects of behavior therapy on regional cerebral blood flow in obsessive-compulsive disorder. Psychiatry Res. 124, 113–120 10.1016/S0925-4927(03)00069-6 14561429

[B195] Nee D. E. Wager T. D. Jonides J. (2007). Interference resolution: insights from a meta-analysis of neuroimaging tasks. Cogn. Affect. Behav. Neurosci. 7, 1–17 10.3758/CABN.7.1.1 17598730

[B196] Nitschke J. B. Sarinopoulos I. Oathes D. J. Johnstone T. Whalen P. J. Davidson R. J. (2009). Anticipatory activation in the amygdala and anterior cingulate in generalized anxiety disorder and prediction of treatment response. Am. J. Psychiatry 166, 302–310 10.1176/appi.ajp.2008.07101682 19122007 PMC2804441

[B197] Ochsner K. N. Silvers J. A. Buhle J. T. (2012). Functional imaging studies of emotion regulation: a synthetic review and evolving model of the cognitive control of emotion. Ann. N.Y. Acad. Sci. 1251, E1–E24 10.1111/j.1749-6632.2012.06751.x 23025352 PMC4133790

[B198] Olesen P. J. Westerberg H. Klingberg T. (2004). Increased prefrontal and parietal activity after training of working memory. Nat. Neurosci. 7, 75–79 10.1038/nn1165 14699419

[B199] Olvet D. M. Hajcak G. (2008). The error-related negativity (ERN) and psychopathology: toward an endophenotype. Clin. Psychol. Rev. 28, 1343–1354 10.1016/j.cpr.2008.07.003 18694617 PMC2615243

[B200] Papageorgiou C. Wells A. (2000). Treatment of recurrent major depression with attention training. Cogn. Behav. Pract. 7, 407–413 10.1016/S1077-7229(00)80051-6

[B201] Paquette V. Lévesque J. Mensour B. Leroux J.-M. Beaudoin G. Bourgouin P. (2003). “Change the mind and you change the brain”: effects of cognitive-behavioral therapy on the neural correlates of spider phobia. Neuroimage 18, 401-409 10.1016/S1053-8119(02)00030-7 12595193

[B202] Paradiso S. Lamberty G. J. Garvey M. J. Robinson R. G. (1997). Cognitive impairment in the euthymic phase of chronic unipolar depression. J. Nerv. Mental Dis. 185, 748–754 9442186 10.1097/00005053-199712000-00005

[B203] Paulus M. P. Rogalsky C. Simmons A. Feinstein J. S. Stein M. B. (2003). Increased activation in the right insula during risk-taking decision making is related to harm avoidance and neuroticism. Neuroimage 19, 1439–1448 10.1016/S1053-8119(03)00251-9 12948701

[B204] Pessoa L. (2008). On the relationship between emotion and cognition. Nat. Rev. Neurosci. 9, 148–158 10.1038/nrn2317 18209732

[B205] Pessoa L. (2009). How do emotion and motivation direct executive control? Trends Cogn. Sci. 13, 160–166 10.1016/j.tics.2009.01.006 19285913 PMC2773442

[B206] Pessoa L. Engelmann J. B. (2010). Embedding reward signals into perception and cognition. Front. Neurosci. 4:17 10.3389/fnins.2010.00017 20859524 PMC2940450

[B207] Phelps E. A. (2006). Emotion and cognition: insights from studies of the human amygdala. Ann. Rev. Psychol. 57, 27–53 10.1146/annurev.psych.56.091103.070234 16318588

[B208] Pittenger C. Duman R. S. (2008). Stress, depression, and neuroplasticity: a convergence of mechanisms. Neuropsychopharmacology 33, 88–109 10.1038/sj.npp.1301574 17851537

[B209] Pizzagalli D. A. Dillon D. G. Bogdan R. Holmes A. J. (2011). Reward and punishment processing in the human brain: clues from affective neuroscience and implications for depression research, in Neuroscience of Decision Making, eds Vartanian O. Mandel D. R. (New York, NY: Psychology Press), 199–220

[B210] Pizzagalli D. A. Holmes A. J. Dillon D. G. Goetz E. L. Birk J. L. Bogdan R. (2009a). Reduced caudate and nucleus accumbens response to rewards in unmedicated subjects with Major Depressive Disorder. Am. J. Psychiatry 166, 702–710 10.1176/appi.ajp.2008.08081201 19411368 PMC2735451

[B211] Pizzagalli D. A. Iosifescu D. Hallett L. A. Ratner K. G. Fava M. (2009b). Reduced hedonic capacity in Major Depressive Disorder: evidence from a probabilistic reward task. J. Psychiatr. Res. 43, 76–87 10.1016/j.jpsychires.2008.03.001 18433774 PMC2637997

[B212] Pizzagalli D. A. Nitschke J. B. Oakes T. R. Hendrick A. M. Horras K. A. Larson C. L. (2002). Brain electrical tomography in depression: the importance of symptom severity, anxiety, and melancholic features. Biol. Psychiatry 52, 73–85 10.1016/S0006-3223(02)01313-6 12113998

[B213] Pizzagalli D. Pascual-Marqui R. D. Nitschke J. B. Oakes T. R. Larson C. L. Abercrombie H. C. (2001). Anterior cingulate activity as a predictor of degree of treatment response in major depression: evidence from brain electrical tomography analysis. Am. J. Psychiatry 158, 405–415 10.1176/appi.ajp.158.3.405 11229981

[B214] Plichta M. M. Schwarz A. J. Grimm O. Morgen K. Mier D. Haddad L. (2012). Test–retest reliability of evoked BOLD signals from a cognitive – emotive fMRI test battery. Neuroimage 60, 1746–1758 t 10.1016/j.neuroimage.2012.01.129 22330316

[B215] Pochon J. B. Levy R. Fossati P. Lehericy S. Poline J. B. Pillon B. (2002). The neural system that bridges reward and cognition in humans: an fMRI study. Proc. Natl. Acad. Sci. U.S.A. 99, 5669–5674 10.1073/pnas.082111099 11960021 PMC122829

[B216] Popov T. Jordanov T. Rockstroh B. Elbert T. Merzenich M. M. Miller G. A. (2011). Specific cognitive training normalizes auditory sensory gating in schizophrenia: a randomized trial. Biol. Psychiatry 69, 465–471 10.1016/j.biopsych.2010.09.028 21092939

[B217] Raes F. Hermans D. Williams J. M. G. Demyttenaere K. Sabbe B. Pieters G. (2005). Reduced specificity of autobiographical memory: a mediator between rumination and ineffective social problem-solving in major depression? J. Affect. Disord. 87, 331–335 10.1016/j.jad.2005.05.004 15979154

[B218] Rector N. A. Beck A. T. (2002). Cognitive therapy for schizophrenia: from conceptualization to intervention. Can. J. Psychiatry 47, 39–48 11873707

[B218a] Rogers M. A. Kasai K. Koji M. Fukuda R. Iwanami A. Nakagome K. (2004). Executive and prefrontal dysfunction in unipolar depression: a review of neuropsychological and imaging evidence. Neurosci. Res. 50, 1–11 10.1016/j.neures.2004.05.003 15288493

[B219] Roseman I. J. (2008). Structure of emotions motivations and emotivations: approach, avoidance, and other tendencies in motivated and emotional behavior, in Handbook of Approach and Avoidance Motivation, ed Elliot A. (New York, NY: Psychology Press), 343–366

[B220] Rushworth M. F. S. Walton M. E. Kennerley S. W. Bannerman D. M. (2004). Action sets and decisions in the medial frontal cortex. Trends Cogn. Sci. 8, 410–417 10.1016/j.tics.2004.07.009 15350242

[B221] Rushworth M. F. Buckley M. J. Behrens T. E. Walton M. E. Bannerman D. M. (2007). Functional organization of the medial frontal cortex. Curr. Opin. Neurobiol. 17, 220 10.1016/j.conb.2007.03.001 17350820

[B222] Salvadore G. Cornwell B. R. Sambataro F. Latov D. Colon-Rosario V. Carver F. (2010). Anterior cingulate desynchronization and functional connectivity with the amygdala during a working memory task predict rapid antidepressant response to ketamine. Neuropsychopharmacology 35, 1415–1422 10.1038/npp.2010.24 20393460 PMC2869391

[B223] Samanez-Larkin G. R. Hollon N. G. Carstensen L. L. Knutson B. (2008). Individual differences in insular sensitivity during loss anticipation predict avoidance learning. Psychol. Sci. 19, 320–323 10.1111/j.1467-9280.2008.02087.x 18399882 PMC2365707

[B224] Sanderson W. C. DiNardo P. A. Rapee R. M. Barlow D. H. (1990). Syndrome comorbidity in patients diagnosed with a DSM-III-R anxiety disorder. J. Abnorm. Psychol. 99, 308–312 10.1037/0021-843X.99.3.308 2212281

[B225] Sass S. M. Heller W. Stewart J. L. Silton R. L. Edgar J. C. Fisher J. E. (2010). Time course of attentional bias in anxiety: emotion and gender specificity. Psychophysiology 47, 247–259 10.1111/j.1469-8986.2009.00926.x 19863758 PMC3073148

[B226] Savine A. C. Beck S. M. Edwards B. G. Chiew K. S. Braver T. S. (2010). Enhancement of cognitive control by approach and avoidance motivational states. Cogn. Emot. 24, 338–356 10.1080/02699930903381564 20390042 PMC2852893

[B227] Saxena S. Brody A. L. Maidment K. M. Dunkin J. J. Colgan M. Alborzian S. (1999). Localized orbitofrontal and subcortical metabolic changes and predictors of response to paroxetine treatment in obsessive-compulsive disorder. Neuropsychopharmacology 21, 683–693 10.1016/S0893-133X(99)00082-2 10633474

[B228] Schwartz J. M. Stoessel P. W. Baxter L. R. Martin K. M. Phelps M. E. (1996). Systematic changes in cerebral glucose metabolic rate after successful behavior modification treatment of obsessive– compulsive disorder. Arch. Gen. Psychiatry 53, 109-113 10.1001/archpsyc.1996.01830020023004 8629886

[B229] See J. MacLeod C. Bridle R. (2009). The reduction of anxiety vulnerability through the modification of attentional bias: a real-world study using a home-based cognitive bias modification procedure. J. Abnorm. Psychol. 118, 65–75 10.1037/a0014377 19222315

[B230] Seedat S. Warwick J. van Heerden B. Hugo C. Zungu-Dirwayi N. Van Kradenburg J. (2004). Single photon emission computed tomography in posttraumatic stress disorder before and after treatment with a selective serotonin reuptake inhibitor. J. Affect. Disord. 80, 45–53 10.1016/S0165-0327(03)00047-8 15094257

[B231] Seminowicz D. A. Mayberg H. S. McIntosh A. R. Goldapple K. Kennedy S. Segal Z. (2004). Limbic-frontal circuitry in major depression: a path modeling metanalysis. Neuroimage 22, 409–418 10.1016/j.neuroimage.2004.01.015 15110034

[B232] Sheline Y. I. Barch D. M. Donnelly J. M. Ollinger J. M. Snyder A. Z. Mintun M. A. (2001). Increased amygdala response to masked emotional faces in depressed subjects resolves with antidepressant treatment: an fMRI study. Biol. Psychiatry 50, 651–658 10.1016/S0006-3223(01)01263-X 11704071

[B233] Siegle G. J. Carter C. Thase M. (2006). Use of fMRI to predict recovery from unipolar depression with cognitive behavior therapy. Am. J. Psychiatry 163, 735–738 10.1176/appi.ajp.163.4.735 16585452

[B234] Siegle G. J. Ghinassi F. Thase M. E. (2007). Neurobehavioral therapies in the 21st century: summary of an emerging field and an extended example of cognitive control training for depression. Cogn. Ther. Res. 31, 235–262 10.1007/s10608-006-9118-6

[B235] Siegle G. J. Thompson W. K. Collier A. Berman S. R. Feldmiller J. Thase M. E. (2012). Toward clinically useful neuroimaging in depression treatment, Arch. Gen. Psychiatry 69, 913–924 10.1001/archgenpsychiatry.2012.65 22945620 PMC3951953

[B236] Silton R. L. Heller W. Engels A. S. Towers D. N. Spielberg J. M. Edgar J. C. (2011). Depression and anxious apprehension distinguish frontocingulate cortical activity during top-down attentional control. J. Abnorm. Psychol. 120, 272–285 10.1037/a0023204 21553941 PMC4406398

[B237] Sloan D. M. Strauss M. E. Quirk S. W. Sajatovic M. (1997). Subjective and expressive emotional responses in depression. J. Affect. Disord. 46, 135–141 10.1016/S0165-0327(97)00097-9 9479617

[B238] Smoski M. J. Felder J. Bizzell J. Green S. R. Ernst M. Lynch T. R. (2009). fMRI of alterations in reward selection, anticipation, and feedback in major depressive disorder. J. Affect. Disord. 118, 69–78 10.1016/j.jad.2009.01.034 19261334 PMC2745481

[B239] Snyder H. R. (2013). Major depressive disorder is associated with broad impairments on neuropsychological measures of executive function: a meta-analysis and review. Psychol. Bull. 139, 81–132 10.1037/a0028727 22642228 PMC3436964

[B240] Somerville L. H. Kim H. Johnstone T. Alexander A. L. Whalen P. J. (2004). Human amygdala responses during presentation of happy and neutral faces: correlations with state anxiety. Biol. Psychiatry 55, 897–903 10.1016/j.biopsych.2004.01.007 15110733

[B241] Spielberg J. M. Heller W. Silton R. L. Stewart J. L. Miller G. A. (2011a). Approach and avoidance profiles distinguish dimensions of anxiety and depression. Cogn. Ther. Res. 35, 359–371 10.1007/s10608-011-9364-0

[B242] Spielberg J. M. Miller G. A. Engels A. S. Herrington J. D. Sutton B. P. Banich M. T. (2011b). Trait approach and avoidance motivation: lateralized neural activity associated with executive function. Neuroimage 54, 661–670 10.1016/j.neuroimage.2010.08.037 20728552 PMC2962704

[B243] Spielberg J. M. Miller G. A. Warren S. L. Engels A. S. Crocker L. D. Banich M. T. (2012a). A brain network instantiating approach and avoidance motivation. Psychophysiology 49, 1200–1214 10.1111/j.1469-8986.2012.01443.x 22845892 PMC4559331

[B244] Spielberg J. M. Miller G. A. Warren S. L. Engels A. S. Crocker L. D. Sutton B. P. (2012b). Trait motivation moderates neural activation associated with goal pursuit. Cogn. Affect. Behav. Neurosci. 12, 308–322 10.3758/s13415-012-0088-8 22460723 PMC3345955

[B245] Spielberg J. M. Stewart J. L. Levin R. L. Miller G. A. Heller W. (2008). Prefrontal cortex, emotion, and approach/withdrawal motivation. Soc. Pers. Psychol. Compass. 2, 135–153 10.1111/j.1751-9004.2007.00064.x 20574551 PMC2889703

[B246] Straube T. Glauer M. Dilger S. Mentzel H.-J. Miltner W. H. R. (2006). Effects of cognitive-behavioral therapy on brain activation in specific phobia. Neuroimage 29, 125-135 10.1016/j.neuroimage.2005.07.007 16087353

[B247] Takeuchi H. Sekiguchi A. Taki Y. Yokoyama S. Yomogida Y. Komuro N. (2010). Training of working memory impacts structural connectivity. J. Neurosci. 30, 3297–3303 10.1523/JNEUROSCI.4611-09.2010 20203189 PMC6634113

[B248] Taylor S. F. Welsh R. C. Wager T. D. Phan K. L. Fitzgerald K. D. Gehring W. J. (2004). A functional neuroimaging study of motivation and executive function. Neuroimage 21, 1045–1054 10.1016/j.neuroimage.2003.10.032 15006672

[B249] Teasdale J. D. Segal Z. V. Williams J. M. G. Ridgeway V. A. Soulsby J. M. Lau M. A. (2000). Prevention of relapse/recurrence in major depression by mindfulness-based cognitive therapy. J. Consult. Clin. Psychol. 68, 615–623 10.1037/0022-006X.68.4.615 10965637

[B250] Tucker D. M. Luu P. Frishkoff G. Quiring J. Poulsen C. (2003). Frontolimbic response to negative feedback in clinical depression. J. Abnorm. Psychol. 112, 667–678 10.1037/0021-843X.112.4.667 14674878

[B251] Vaidya V. A. Duman R. S. (2001). Depression - emerging insights from neurobiology. Br. Med. Bull. 57, 61–79 10.1093/bmb/57.1.61 11719924

[B252] Vasic N. Walter H. Sambataro F. Wolf R. C. (2009). Aberrant functional connectivity of dorsolateral prefrontal and cingulate networks in patients with major depression during working memory processing. Psychol. Med. 39, 977–987 10.1017/S0033291708004443 18845009

[B253] Vermetten E. Vythilingam M. Southwick S. M. Charney D. S. Bremner J. D. (2003). Long-term treatment with paroxetine increases verbal declarative memory and hippocampal volume in posttraumatic stress disorder. Biol. Psychiatry 54:693 10.1016/S0006-3223(03)00634-6 14512209 PMC3233762

[B254] Vogt A. Kappos L. Calabrese P. Stöcklin M. Gschwind L. Opwis K. (2009). Working memory training in patients with multiple sclerosis - comparison of two different training schedules. Restorat. Neurol. Neurosci. 27, 225–235 10.3233/RNN-2009-0473 19531877

[B255] Wacker J. Dillon D. G. Pizzagalli D. A. (2009). The role of the nucleus accumbens and rostral anterior cingulate cortex in anhedonia: integration of resting EEG, fMRI, and volumetric techniques. Neuroimage 46, 327–337 10.1016/j.neuroimage.2009.01.058 19457367 PMC2686061

[B256] Wager T. D. Jonides J. Reading S. (2004). Neuroimaging studies of shifting attention: a meta-analysis. Neuroimage 22, 1679–1693 10.1016/j.neuroimage.2004.03.052 15275924

[B257] Wager T. D. Smith E. E. (2003). Neuroimaging studies of working memory: a meta-analysis. Cogn. Affect. Behav. Neurosci. 3, 255–274 10.3758/CABN.3.4.255 15040547

[B258] Watkins E. R. Baeyens C. B. Read R. (2009). Concreteness training reduces dysphoria: proof-of-principle for repeated cognitive bias modification in depression. J. Abnorm. Psychol. 118, 55–64 10.1037/a0013642 19222314

[B259] Whalen P. J. Bush G. McNally R. J. Wilhelm S. McInerney S. C. Jenike M. A. (1998). The emotional counting Stroop paradigm: a functional magnetic resonance imaging probe of the anterior cingulate affective division. Biol. Psychiatry 44, 1219–1228 10.1016/S0006-3223(98)00251-0 9861465

[B260] Whalen P. J. Johnstone T. Somerville L. H. Nitschke J. B. Polis S. Alexander A. L. (2008). A functional magnetic resonance imaging predictor of treatment response to venlafaxine in generalized anxiety disorder. Biol. Psychiatry 63, 858 10.1016/j.biopsych.2007.08.019 17964548 PMC2654286

[B261] Williams J. M. G. Barnhofer T. Crane C. Herman D. Raes F. Watkins E. (2007). Autobiographical memory specificity and emotional disorder. Psychol. Bull. 133, 122–148 10.1037/0033-2909.133.1.122 17201573 PMC2834574

[B262] Williams J. M. G. Watts F. N. MacLeod C. Mathews A. (1997). Cognitive Psychology and Emotional Disorders. 2nd Edn Chichester, UK: Wiley

[B263] Williams R. A. Hagerty B. M. Cimprich B. Therrien B. Bay E. Oe H. (2000). Changes in directed attention and short-term memory in depression. J. Psychiatr. Res. 34, 227–238 10.1016/S0022-3956(00)00012-1 10867118

[B264] Wilson E. MacLeod C. Mathews A. Rutherford E. M. (2006). The causal role of interpretive bias in anxiety reactivity. J. Abnorm. Psychol. 115, 103–111 10.1037/0021-843X.115.1.103 16492101

[B265] Yee C. M. (1995). Implications of the resource allocation model for mood disorders, in The Behavioral High-Risk Paradigm in Psychopathology, ed Miller G. A. (New York, NY: Springer-Verlag), 271–288 10.1007/978-1-4612-4234-5_10

[B266] Yee C. M. Miller G. A. (1994). A dual-task analysis of resource allocation in dysthymia and anhedonia. J. Abnorm. Psychol. 103, 625–636 10.1037/0021-843X.103.4.625 7822563

[B267] Young K. D. Erickson K. Nugent A. C. Fromm S. J. Mallinger A. G. Furey M. L. (2012). Functional anatomy of autobiographical memory recall deficits in depression. Psychol. Med. 1, 1–13 10.1017/S0033291711001371 21798113 PMC3226869

[B268] Zinbarg R. E. Yoon K. L. (2008). RST and clinical disorders: anxiety and depression, in The Reinforcement Sensitivity Theory of Personality, ed Corr P. J. (Cambridge, UK: Cambridge University Press), 360–397 10.1017/CBO9780511819384.013

